# Characterization of the molecular mechanisms underlying azithromycin‐induced cardiotoxicity using human‐induced pluripotent stem cell‐derived cardiomyocytes

**DOI:** 10.1002/ctm2.549

**Published:** 2021-09-16

**Authors:** Xiaochen Wang, Ziwei Pan, Jue Wang, Hongkun Wang, Hangping Fan, Tingyu Gong, Qiming Sun, Ye Feng, Ping Liang

**Affiliations:** ^1^ Key Laboratory of combined Multi‐organ Transplantation Ministry of Public Health the First Affiliated Hospital Zhejiang University School of Medicine Hangzhou Zhejiang 310003 China; ^2^ Institute of Translational Medicine Zhejiang University Hangzhou 310029 China; ^3^ Sir Run Run Shaw Hospital Zhejiang University School of Medicine Hangzhou 310016 China; ^4^ Department of Biochemistry Department of Cardiology of Second Affiliated Hospital Zhejiang University School of Medicine Hangzhou 310058 China

**Keywords:** autophagy, azithromycin, cardiotoxicity, iPSC‐CMs, lysosome, QT interval

Dear Editor,

We performed a comprehensive study to characterize the molecular mechanisms underlying azithromycin (AZM)‐induced cardiotoxicity (AIC) using human‐induced pluripotent stem cell‐derived cardiomyocytes (iPSC‐CMs). Electrophysiologically, high‐concentration AZM causes accelerated beating rate and dramatically shortened QT in the absence of proarrhythmic risk. Morphologically, high‐concentration AZM interferes with lysosomal activity to impair autophagy flux and autophagosome maturation. The futile and excessive autophagosome formation and accumulation confers vacuole formation, sarcomeric damage, and cardiomyocyte death. Our study uncovered a novel molecular mechanism underlying AIC, and reducing the accumulation of autophagosomes may offer a novel therapeutic strategy for potential AZM‐induced cardiovascular risk.

AZM is one of the most frequently used antibiotics linked to an increased risk of fatal ventricular arrhythmias and cardiovascular death.^1^ Results of AZM‐associated cardiovascular risk in existing retrospective studies were discordant, and underlying mechanisms remain unclear.^2^, ^3^, ^4^ Three healthy iPSC‐CM lines were utilized to investigate AIC, such as arrhythmias, reduced cell viability and morphological damage (Figure S1‐S2). Field potentials (FPs) and action potentials were recorded from iPSC‐CMs by multi‐electrode array and patch clamp, and baseline electrophysiological parameters were comparable between three iPSC‐CM lines (Figure S3‐S4). Dimethyl sulfoxide (DMSO) and moxifloxacin (MXF) were firstly tested as negative and positive drugs. As expected, DMSO showed negligible effect on FPs, whereas MXF caused prominent prolongation of FP duration (FPD) (Figure 1A–D, Table S1). Having established a stable drug testing platform, we next assessed the acute effects of AZM on electrophysiology (Figure S5). Strikingly, AZM resulted in strong effects on FPs in a concentration‐dependent manner (Figure 1E). Starting from 3 μM, acute AZM treatment caused significantly increased beating rate and shortened FPD (Figure 1F, Table S1). We observed no arrhythmic activity even at the maximal tested concentration of AZM. Consistent with previous studies,^5^, ^6^ we observed that 100 μM AZM caused approximately 20% inhibition of human ether‐à‐go‐go‐related gene (hERG) currents, indicating a minimal effect on hERG (Figure S6). Ca^2+^ currents isolated from 10 and 30 μM AZM‐treated iPSC‐CMs were significantly reduced, and steady‐state activation (SSA) curve in 30 μM AZM‐treated iPSC‐CMs was significantly right‐shifted (Figure 1G‐L, Table S2). Moreover, acutely treated with 30 μM AZM, Na^+^ current density was significantly decreased, and steady‐state inactivation (SSI) was significantly left‐shifted (Figure 1M‐R, Table S2). No significant change was observed in K^+^ current recordings (Figure S7).

**FIGURE 1:**
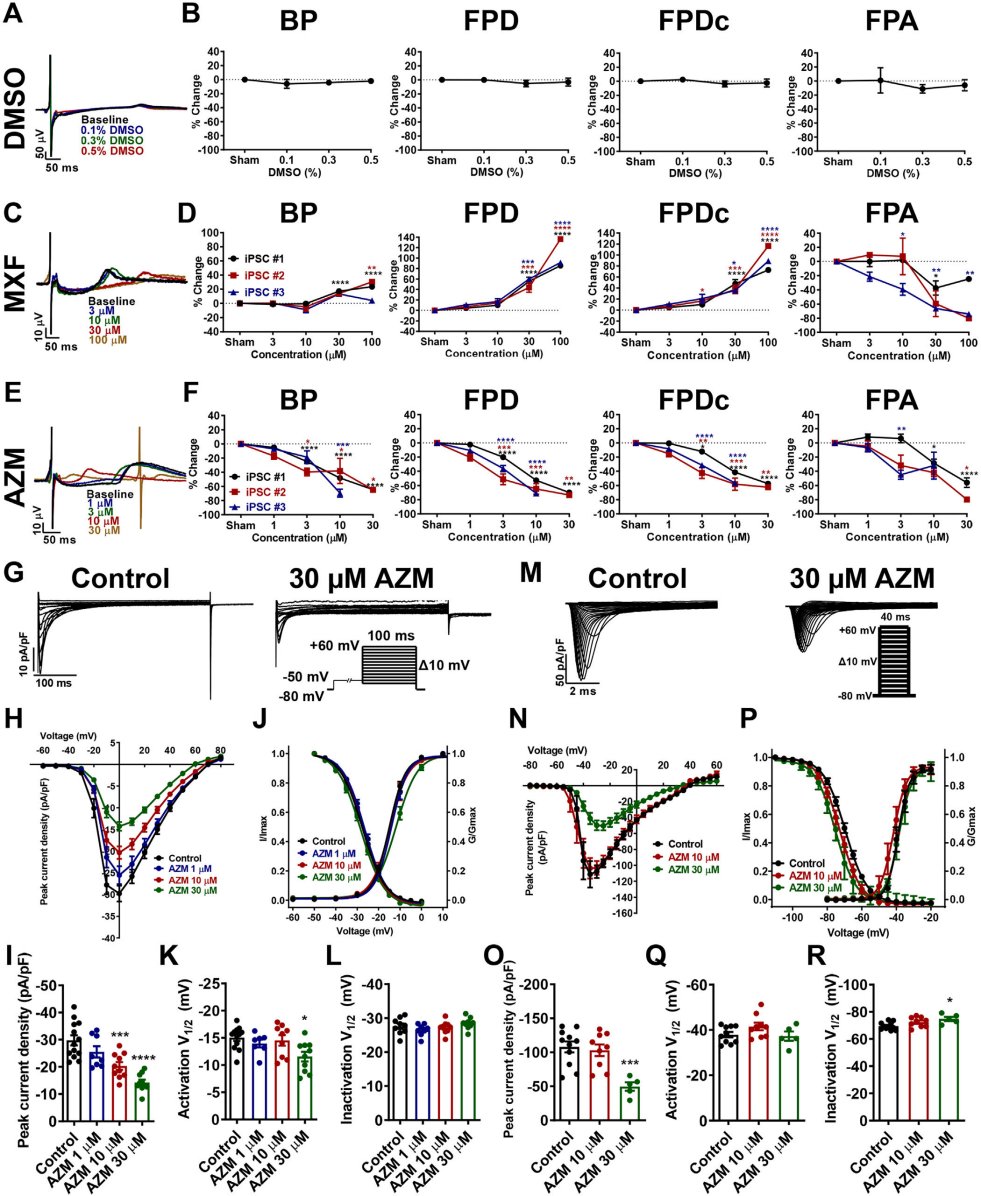
**Electrophysiological effects of acute azithromycin (AZM) in induced pluripotent stem cell‐derived cardiomyocytes (iPSC‐CMs)**. (**A**) Representative field potential (FP) tracings recorded from iPSC‐CMs with acute treatment of 0, 0.1%, 0.3%, and 0.5% dimethyl sulfoxide (DMSO), respectively. (**B**) Acute effects of DMSO on beating period (BP), field potential duration (FPD), corrected field potential duration (FPDc), and field potential amplitude (FPA). (**C**) Representative FP tracings recorded from iPSC‐CMs with acute treatment of 0, 3, 10, 30, and 100 μM moxifloxacin (MXF), respectively. (**D**) Acute effects of MXF on BP, FPD, FPDc, and FPA. Prominent prolongation of FPD and FPDc was observed in response to acute treatment of MXF in a concentration‐dependent manner. BP was significantly increased at 30 and 100 μM, suggesting a slower effect on beating rate induced by MXF. Note that 30 and 100 μM MXF had largely decreased effect on FPA. Mycoytes were derived from three different iPSC lines. ^*^ *p *< 0.05, ^**^ *p *< 0.01, ^***^ *p *< 0.001, and ^****^ *p *< 0.0001. (**E**) Representative FP tracings recorded from iPSC‐CMs with acute treatment of 0, 1, 3, 10, and 30 μM AZM, respectively. (**F**) Acute effects of AZM on BP, FPD, FPDc, and FPA. Beating rate, reflected by BP, was significantly increased started at the concentration of 3 μM AZM. Starting from 3 μM, acute treatment of AZM gave rise to markedly shortened FPD and FPDc. The FPA was significantly decreased by 10 and 30 μM AZM treatment. Mycoytes were derived from three different iPSC lines. ^*^ *p *< 0.05, ^**^ *p *< 0.01, ^***^ *p *< 0.001, and ^****^ *p *< 0.0001. (**G**) Representative Ca^2+^ current tracings isolated from control iPSC‐CMs and iPSC‐CMs with acute treatment of 30 μM AZM. Myocytes were derived from iPSC #3. (**H**) Comparison of Ca^2+^ current‐voltage relationship curve (IV curve) between control iPSC‐CMs and iPSC‐CMs with acute treatment of 1, 10, and 30 μM AZM. (**I**) Bar graph to compare peak Ca^2+^ current density at 0 mV between different groups in H. *n* = 8–13. We observed concentration‐dependent inhibition of Ca^2+^ currents in AZM‐treated iPSC‐CMs, and significant changes were achieved at 10 and 30 μM. ^***^ *p *< 0.001 and ^****^ *p *< 0.0001. (**J**) Comparison of steady‐state activation (SSA) and steady‐state inactivation (SSI) of Ca^2+^ current between control iPSC‐CMs and iPSC‐CMs with acute treatment of 1, 10, and 30 μM AZM. (**K** and **L**) Bar graphs to compare V_1/2_ of SSA and SSI of Ca^2+^ current between different groups in J. SSA, *n* = 7–12; SSI, *n* = 8–9. SSA curve of Ca^2+^ currents in 30 μM AZM‐treated iPSC‐CMs was significantly right‐shifted as compared to controls, whereas SSI curves of Ca^2+^ currents stayed unchanged between control and AZM‐treated iPSC‐CMs. ^*^ *p *< 0.05. (**M**) Representative Na^+^ current tracings isolated from control iPSC‐CMs and iPSC‐CMs with acute treatment of 30 μM AZM. (**N**) Comparison of Na^+^ IV curve between control iPSC‐CMs and iPSC‐CMs with acute treatment of 10 and 30 μM AZM. Myocytes were derived from iPSC #3. (**O**) Bar graph to compare peak Na^+^ current density at −30 mV between different groups in N. *n* = 5‐11. Slight reduction of Na^+^ currents was noted by acute treatment of 10 μM AZM, and this change turned to be statistically significant upon 30 μM AZM treatment. ^***^ *p *< 0.001. (**P**) Comparison of SSA and SSI of Na^+^ current between control iPSC‐CMs and iPSC‐CMs with acute treatment of 10 and 30 μM AZM. (**Q** and **R**) Bar graphs to compare V_1/2_ of SSA and SSI of Na^+^ current between different groups in P. SSA, *n* = 5–10; SSI, *n* = 5–11. SSA curves of Na^+^ currents were comparable between control and AZM‐treated iPSC‐CMs. However, SSI curve of Na^+^ currents in 30 μM AZM‐treated iPSC‐CMs was significantly left‐shifted. ^*^ *p *< 0.05

Electrophysiological effects of chronic AZM in iPSC‐CMs were also assessed. DMSO caused slight changes of FPs on day 1, which were diminished along the treatment and recovered to baseline on day 5 (Figure 2A,B, Table S3). Consistent with the observations of acute AZM treatment, dramatic changes of FPs were seen starting on day 1, resulting in accelerated beating rate and FPD shortening, which stayed constant from day 2 to day 5 postinduction (Figure 2C,D, Table S3). No arrhythmic activities were detected throughout 5‐day recordings. Ca^2+^ currents were largely reduced in AZM‐treated iPSC‐CMs while Na^+^ and K^+^ currents remained unchanged (Figure 2E‐P, Figure S8, Table S4).

**FIGURE 2:**
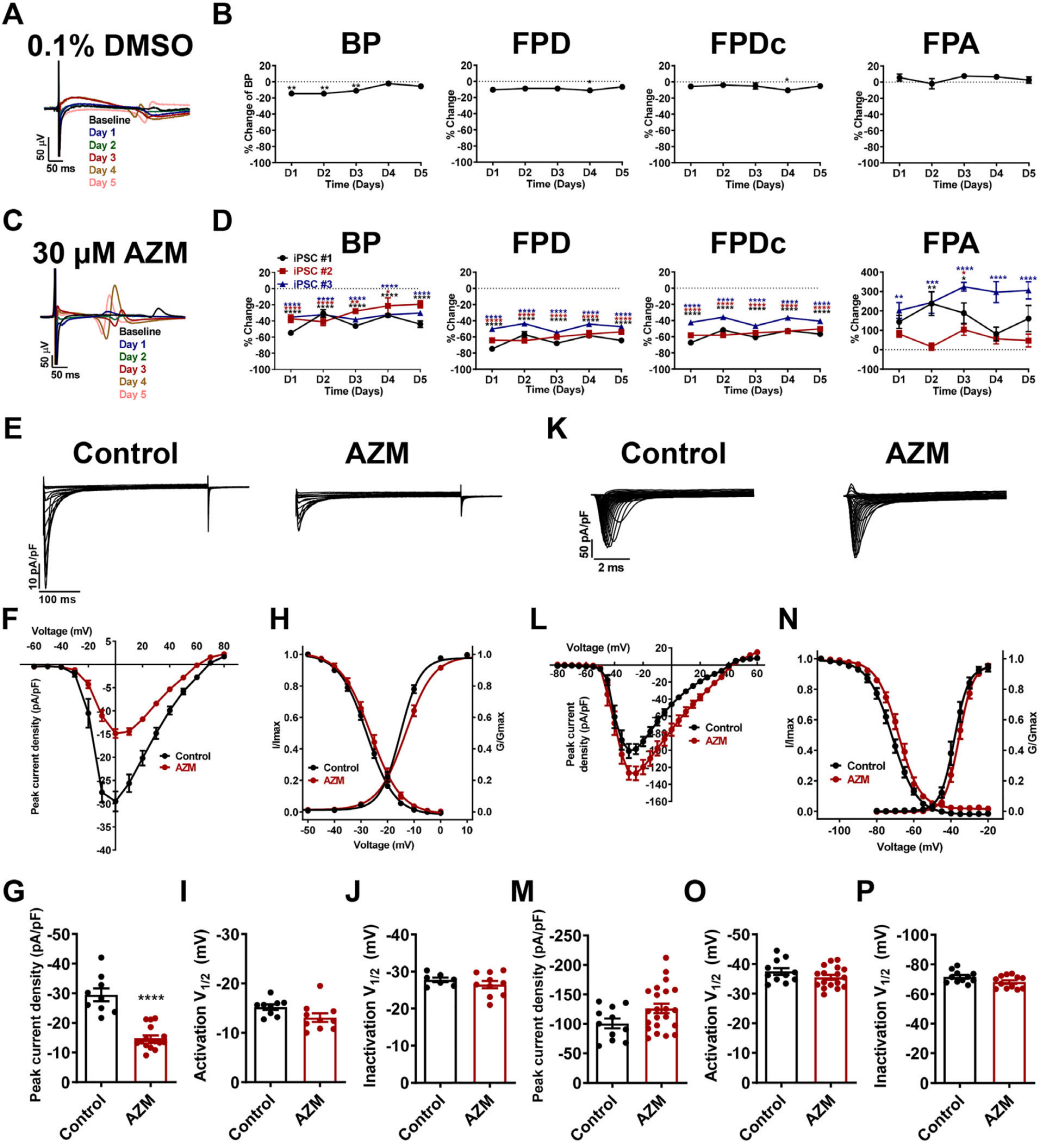
**Electrophysiological effects of chronic AZM in iPSC‐CMs**. (**A**) Representative FP tracings recorded from iPSC‐CMs with chronic treatment of 0.1% DMSO on day 0–5. (**B**) Chronic effects of 0.1% DMSO on BP, FPD, FPDc, and FPA. ^*^ *p *< 0.05 and ^**^ *p *< 0.01. (**C**) Representative FP tracings recorded from iPSC‐CMs with chronic treatment of 30 μM AZM on day 0–5. (**D**) Chronic effects of 30 μM AZM on BP, FPD, FPDc, and FPA. In line with the observations of acute effects by AZM, dramatic changes of FPs were seen starting on day 1, resulting in accelerated beating rate and shortened FPD and FPDc, which stayed constant from day 2 to day 5 postinduction. Conversely, we observed increased effect on FPA by chronic treatment of AZM, which was significantly decreased in acute AZM test. Mycoytes were derived from three different iPSC lines. ^*^ *p *< 0.05, ^**^ *p *< 0.01, ^***^ *p *< 0.001, and ^****^ *p *< 0.0001. (**E**) Representative Ca^2+^ current tracings isolated from control and AZM (30 μM, 5 days)‐treated iPSC‐CMs. Myocytes were derived from iPSC #3. (**F**) Comparison of Ca^2+^ IV curve between control and AZM (30 μM, 5 days)‐treated iPSC‐CMs. (**G**) Bar graph to compare peak Ca^2+^ current density at 0 mV between the two groups in F. *n* = 9–16. ^****^ *p *< 0.0001. (**H**) Comparison of SSA and SSI of Ca^2+^ current between control and AZM (30 μM, 5 days)‐treated iPSC‐CMs. (**I** and **J**) Bar graphs to compare V_1/2_ of SSA and SSI of Na^+^ current between the two groups in H. SSA, *n* = 9–10; SSI, *n* = 7–10. (**K**) Representative Na^+^ current tracings isolated from control and AZM (30 μM, 5 days)‐treated iPSC‐CMs. Myocytes were derived from iPSC #3. (**L**) Comparison of Na^+^ IV curve between control and AZM (30 μM, 5 days)‐treated iPSC‐CMs. (**M**) Bar graph to compare peak Na^+^ current density at −30 mV between the two groups in L. *n* = 11–22. (**N**) Comparison of SSA and SSI of Na^+^ current between control and AZM (30 μM, 5 days)‐treated iPSC‐CMs. (**O** and **P**) Bar graphs to compare V_1/2_ of SSA and SSI of Na^+^ current between the two groups in N. SSA, *n* = 11–17; SSI, *n* = 11–12

We next sought to determine whether AZM can induce cell death and morphological changes. Starting from 10 μM, we observed cytotoxic effects in AZM‐treated iPSC‐CMs in a concentration‐ and time‐dependent manner (Figure 3A, FigureS9). Significantly reduced cell viability was noted in three iPSC‐CM lines starting from 30 μM AZM (Figure 3B). We thus selected the 5‐day 30 μM AZM treatment plan for downstream assays. Importantly, we observed severe morphological phenotypes in AZM‐treated iPSC‐CMs, including formation of intracellular vacuoles and sarcomeric damage (Figure 3C,D, Figures S10‐S12). Reactive oxygen species (ROS) was significantly increased in AZM‐treated iPSC‐CMs, whereas anti‐oxidant markedly reversed the ROS amount elevation and ameliorated AZM‐induced cell viability reduction (Figure S13).

**FIGURE 3:**
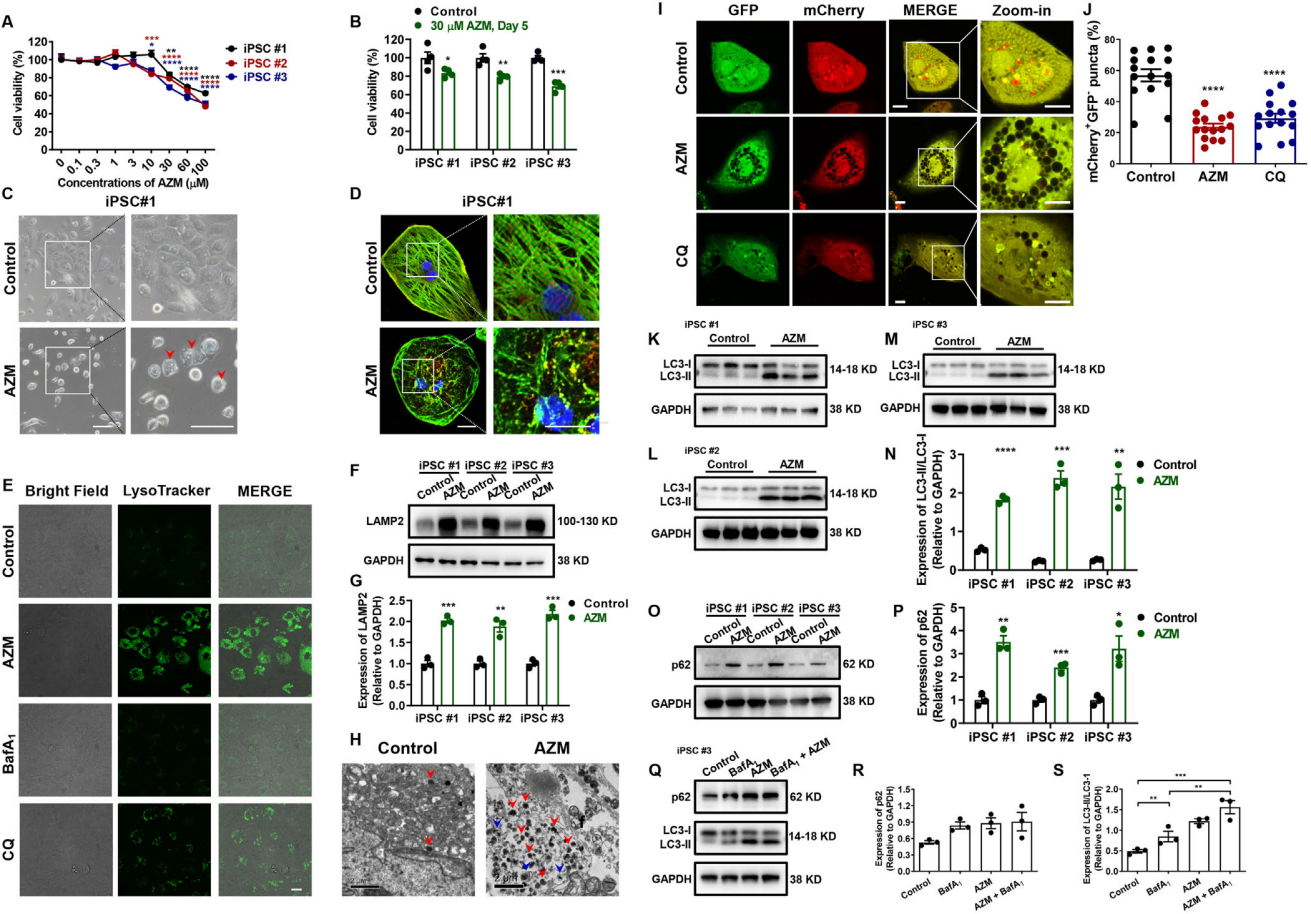
**AZM causes cell death and morphological changes by interfering with lysosomes to impair autophagy flux and autophagosome maturation in iPSC‐CMs**. (**A**) Line graph to compare the cell viability by CCK8 assay between control and AZM‐treated myocytes derived from three different iPSC lines at different concentrations. *n* = 4. ^*^ *p *< 0.05, ^**^ *p *< 0.01, ^***^ *p *< 0.001, and ^****^ *p *< 0.0001. (**B**) Bar graph to compare the cell viability between control and 30 μM AZM‐treated myocytes derived from three different iPSC lines. *n* = 4. ^*^ *p *< 0.05, ^**^ *p *< 0.01, and ^***^ *p *< 0.001. (**C**) Representative morphology of control and 30 μM AZM‐treated myocytes derived from iPSC #1. Red arrows indicate the vacuole formation. Scale bar = 100 μm. (**D**) Immunofluorescent staining of control and 30 μM AZM‐treated myocytes derived from iPSC #1 using TNNT2 (green) and α‐actinin (red). DAPI indicates nuclear staining (blue). Enlarged views showing cardiac sarcomeres in the two groups. Scale bar = 20 μm. (**E**) Representative confocal images of brightfield and LysoTracker staining in control iPSC‐CMs, AZM‐treated iPSC‐CMs, iPSC‐CMs treated with Bafilomycin A_1_ (BafA_1_) (10 nM, 12 h), and iPSC‐CMs treated with chloroquine (CQ) (30 μM, 12 h), respectively. Myocytes were derived from iPSC #3. Scale bar = 20 μm. As positive controls, iPSC‐CMs were treated with CQ or BafA_1_, which are both lysosomal inhibitors. Treatment of BafA_1_ expectedly decreased the acidity of lysosomes by inhibiting H^+^‐ATPase and led to a decrease of LysoTracker puncta staining. However, such effect was not seen in CQ‐treated iPSC‐CMs, which exhibited enhanced LysoTracker puncta staining as comapared to controls. Similar with CQ, AZM treatment resulted in markedly enlarged, dilatate and accumulated lysosomes. (**F**) Western blot analysis of the LAMP2 expression in control and AZM‐treated myocytes from three different iPSC lines. (**G**) Bar graph to compare the lysosomal associated membrane protein 2 (LAMP2) expression between the two groups. *n* = 3. ^**^ *p *< 0.01 and ^***^ *p *< 0.001. (**H**) Representative transmission electron microscope (TEM) images of control and AZM‐treated myocytes derived from iPSC #3. Scale bar = 2 μm. Red and blue arrows indicate lysosomes and autophagosomes, respectively. (**I**) Representative confocal images of mCherry‐GFP‐LC3 expressed in control, AZM‐treated and CQ‐treated iPSC‐CMs. Red fluorescence (mCherry^+^GFP^‐^) indicates autolysosomes whereas yellow fluorescence (mCherry^+^GFP^+^) indicates autophagosomes. Myocytes were derived from iPSC #3. Scale bar = 10 μm. (**J**) Bar graph to compare the percentage of mCherry^+^GFP^‐^ puncta in control, AZM‐treated or CQ‐treated iPSC‐CMs. *n* = 15. ^****^ *p *< 0.0001. (**K‐M**) Western blot analysis of the microtubule associated protein 1 light chain (LC3)‐II/LC3‐I expression in control and AZM‐treated myocytes from three different iPSC lines. (**N**) Bar graph to compare the LC3‐II/LC3‐I expression between control and AZM‐treated iPSC‐CMs. *n* = 3. ^**^ *p *< 0.01 and ^***^ *p *< 0.001. (**O**) Western blot analysis of the p62 expression in control and AZM‐treated myocytes from three different iPSC lines. (**P**) Bar graph to compare the p62 expression between control and AZM‐treated myocytes from three different iPSC lines. *n* = 3. ^*^ *p *< 0.05, ^**^ *p *< 0.01, and ^***^ *p *< 0.001. (**Q**) Western blot analysis of the p62 and LC3‐II/LC3‐I expression in control iPSC‐CMs, BafA_1_‐treated iPSC‐CMs, AZM‐treated iPSC‐CMs, and iPSC‐CMs treated with AZM and BafA_1_. Myocytes were derived from iPSC #3. (**R** and **S**) Bar graphs to compare the p62 and LC3‐II/LC3‐I expression between different groups in Q. *n* = 3. ^**^ *p *< 0.01 and ^***^ *p *< 0.001

To further elucidate the underlying mechanisms, we performed RNA sequencing of control and AZM‐treated iPSC‐CMs, which demonstrated that differentially expressing genes (DEGs) were enriched in lysosome pathway (Figures S14‐S15). To test if AZM‐induced cytotoxicity correlates with lysosomes, we performed live‐cell confocal microscopy using pH‐sensitive LysoTracker as a specific dye for lysosomes.^7^, ^8^ Similar with chloroquine (CQ), AZM caused markedly enlarged, dilatate, and accumulated lysosomes (Figure 3E). Expression of lysosomal associated membrane protein 2 (LAMP2), a specific marker for lysosomes, was significantly enhanced in AZM‐treated iPSC‐CMs (Figure 3F,G), whereas cathespin D expression was unchanged (Figure S16). Transmission electron microscope exhibited greatly increased number of lysosomes and autophagosomes in AZM‐treated iPSC‐CMs (Figure 3H). Given that the lysosome is a key factor in autophagy, we next investigated if AZM‐induced lysosomal changes may affect autophagy flux.^8^ We observed a high proportion of red puncta in untreated cells, indicating the basal state of the autolysosomes. In contrast, the proportion of red puncta was significantly lower in AZM‐ or CQ‐treated iPSC‐CMs (Figure 3I,J).

Moving forward, we sought to determine how AZM affects autophagy. AZM‐treated iPSC‐CMs demonstrated a significantly increased protein expression of microtubule associated protein 1 light chain (LC3)‐II/LC3‐I and p62, pointing to suppressed late‐stage autophagy (Figure 3K‐P). Moreover, iPSC‐CMs were treated with bafilomycin A_1_ (BafA_1_) to block fusion of autophagosomes with lysosomes.^9^ BafA_1_ did not affect AZM‐induced p62 and LC3‐II/LC3‐I upregulation, further indicating that AZM causes impaired autophagy flux and autophagosome maturation (Figure 3Q‐S, Figure S17). However, expression of beclin 1 remained unchanged (Figure S18).

We next assessed whether intervention of autophagy may rescue AZM‐induced cytotoxic phenotypes in iPSC‐CMs.^10^ Induction of autophagy by specific mTOR inhibitor Torin showed no rescuing effects (Figure 4A‐C, G, Figure S19‐S20). In contrast, inhibition of early‐stage autophagy by ULK1 inhibitor MRT68921 significantly restored AZM‐induced p62 and LC3‐II/LC3‐I upregulation, and effectively rescued the reduced cell viability phenotype, although failed to rescue vacuole formation and enhanced LAMP2 expression (Figure 4D‐F, H‐M, Figure S21‐S22). Interestingly, we found that removal of AZM‐containing medium significantly rescued AZM‐induced cytotoxic phenotypes, exhibiting not only restored cell viability, but also suppressed vacuole formation and rescued lysosomes (Figure 4N,O).

**FIGURE 4:**
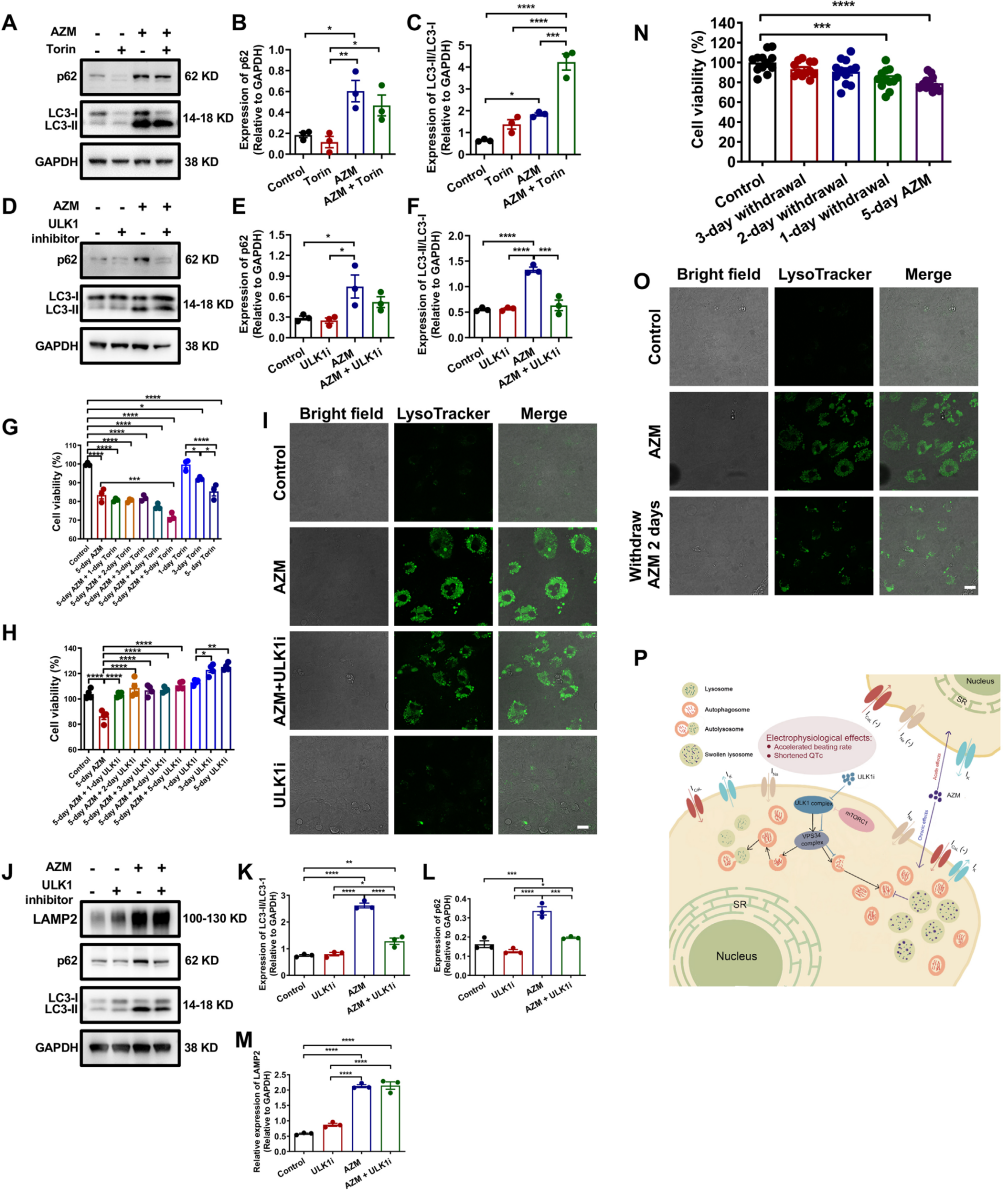
**Rescuing AZM‐induced cytotoxicity by inhibition of early‐stage autophagy and medium removal in iPSC‐CMs**. (**A**) Western blot analysis of p62 and LC3‐II/LC3‐I expression in control and AZM‐treated myocytes derived from iPSC #3 with or without Torin (2.5 nM, 5 days). (**B** and **C**) Bar graphs to compare p62 and LC3‐II/LC3‐I expression between different groups in A. *n* = 3. Torin did not alleviate AZM‐induced upregulation of p62 and even exacerbated the LC3‐II/LC3‐I upregulation phenotype caused by AZM. ^*^ *p *< 0.05, ^**^ *p *< 0.01, ^***^ *p *< 0.001, and ^****^ *p *< 0.0001. (**D**) Western blot analysis of the p62 and LC3‐II/LC3‐I expression in control and AZM‐treated myocytes derived from iPSC #3 with or without MRT68921 (1 μM, 5 days), an ULK1 inhibitor (ULK1i). (**E** and **F**) Bar graphs to compare p62 and LC3‐II/LC3‐I expression between different groups in D. *n* = 3. ^*^ *p *< 0.05, ^***^ *p *< 0.001, and ^****^ *p *< 0.0001. (**G**) Bar graph to compare the cell viability between control and AZM‐treated myocytes derived from iPSC #1 with or without Torin. *n* = 4. ^*^ *p *< 0.05, ^**^ *p *< 0.01, and ^****^ *p *< 0.0001. (**H**) Bar graph to compare the cell viability between control and AZM‐treated myocytes derived from iPSC #1 with or without ULK1i. *n* = 4. 5‐day treatment of ULK1i effectively rescued the reduced cell viability phenotype in AZM‐treated iPSC‐CMs, whereas Torin did not show any rescuing effect and even exacerbated the cytotoxic phenotypes. ^*^ *p *< 0.05, ^***^ *p *< 0.001, and ^****^ *p *< 0.0001. (**I**) Representative confocal images of brightfield and LysoTracker staining in control iPSC‐CMs, AZM‐treated iPSC‐CMs, ULK1i‐treated iPSC‐CMs, or iPSC‐CMs treated with AZM and ULK1i. Myocytes were derived from iPSC #1. Scale bar = 20 μm. (**J**) Western blot analysis of LAMP2, p62 and LC3‐II/LC3‐I expression in control and AZM‐treated myocytes derived from iPSC #1 with or without ULK1i. (**K‐M**) Bar graphs to compare LAMP2, p62 and LC3‐II/LC3‐I expression between different groups in J. *n* = 3. ^*^ *p *< 0.05, ^**^ *p *< 0.01, ^***^ *p *< 0.001, and ^****^ *p *< 0.0001. (**N**) Bar graph to compare the cell viability between control iPSC‐CMs, AZM‐treated iPSC‐CMs, and AZM‐treated iPSC‐CMs with 1‐day, 2‐day or 3‐day AZM withdrawal. Myocytes were derived from iPSC #1. *n* = 12. ^***^ *p *< 0.001 and ^****^ *p *< 0.0001. (**O**) Representative confocal images of brightfield and LysoTracker staining in control iPSC‐CMs, AZM‐treated iPSC‐CMs, AZM‐treated iPSC‐CMs with 2‐day AZM withdrawal. Myocytes were derived from iPSC #1. Scale bar = 20 μm. Removal of AZM‐containing medium for 2 days significantly rescued AZM‐induced cytotoxic phenotypes in iPSC‐CMs. (**P**) Proposed work model of AZM‐induced cardiotoxicity. Electrophysiologically, high‐concentration AZM causes accelerated beating rate and dramatically shortened QTc in the absence of proarrhythmic risk in healthy control populations by acutely suppressing Na^+^ and Ca^2+^ channels, or chronically suppressing Ca^2+^ channel. Morphologically, high‐concentration AZM interferes with lysosomal activity to impair autophagy flux and autophagosome maturation. The futile and excessive autophagosome formation and accumulation confers vacuole formation, sarcomeric damage, and cardiomyocyte death. Inhibition of early‐stage autophagy to alleviate the burden of autophagosome accumulation by ULK1i may partially rescue the deleterious phenotypes

In conclusion, low‐concentration AZM is electrophysiologically and morphologically noncardiotoxic, whereas high‐concentration AZM may cause dramatic QT shortening, cardiomyocyte death, and structural damage but has no proarrhythmic risk in healthy control populations. Our findings suggest that AZM can be prescribed when warranted, but attention should be paid to high‐risk patients with preexisting comorbidities (Figure 4P).

## CONFLICT OF INTEREST

The authors declare that there is no conflict of interest.

## AUTHOR CONTRIBUTIONS

P.L., Y.F. and Q.S. designed and supervised the study. X.W., Z.P., J.W., H.W., H.F., T.G. and Y.F. performed the experiments and analyzed data. Z.P. and P.L. wrote the manuscript.

## DATA AVAILABILITY STATEMENT

The data that support the findings of the study are available from the corresponding author upon reasonable request.

## Supporting information



Supporting Information
