# Supplementary material for: Characterization of the molecular mechanisms underlying azithromycin‐induced cardiotoxicity using human‐induced pluripotent stem cell‐derived cardiomyocytes
Source: Clin Transl Med. 2021 Sep 16;11(9):e549. doi: 10.1002/ctm2.549 (PMC8444559; doi:10.1002/ctm2.549)
Supplement: Supplementary file 1 — Supporting Information [file CTM2-11-e549-s001.pdf]

Supplemental Material

SUPPLEMENTAL MATERIALS AND METHODS

Culture and maintenance of iPSCs

iPSCs were cultured in feeder-free mTeSR1 (STEMCELL Technologies) media on matrigel-coated (Corning) plates at 37°C with 5% (vol/vol) CO_2. The media were daily changed and cells were passaged every 3-4 days using Accutase (STEMCELL Technologies).

Alkaline phosphatase staining

Alkaline phosphatase (ALP) staining was performed using the VECTOR Blue Alkaline Phosphatase Substrate Kit (Vector Laboratories) following the manufacturer's instructions.

Cardiac differentiation

The iPSCs were differentiated into CMs using a 2D monolayer differentiation protocol. Briefly, ~10^5 undifferentiated cells were dissociated and re-plated into matrigel-coated 6-well plates. Cells were cultured and expanded to 85% cell confluence, and then treated for 2 days with 6 µM CHIR99021 (Axon Medchem) in RPMI and B-27 supplement minus insulin (RPMI+B27-Insulin) (Gibco) to activate Wnt signaling pathway. On day 2, cells were placed in RPMI+B27-Insulin with CHIR99021 removal. On days 3-4, cells were treated with 5 µM IWR-1 (Merck) to inhibit Wnt signaling pathway. On day 5-6, cells were removed from IWR-1 treatment and placed in RPMI+B27-Insulin. From day 7 onwards, cells were placed and cultured in RPMI and B-27 supplement with insulin (RPMI+B27+Insulin) (Gibco) until beating was observed. Cells were glucose-starved for 3 days with

RPMI+B27+Insulin for the purification. The iPSC-CMs of day 30-40 after cardiac differentiation were utilized for downstream functional assays in this study.

FACS analysis of iPSC-CMs

Monolayer CMs were dissociated into single cells using 0.25% Tripsin-EDTA (Gibco) for 5 minutes at 37°C. Cells were pelleted and fixed with 4% paraformaldehyde (PFA) (Sangon Biotech) for 10 minutes on ice. Every step was washed with PBS (Sangon Biotech) before sample centrifugation. Cells were stained with TNNT2 (Abcam) at 4°C, and FITC-conjugated goat anti-mouse IgG antibody (Invitrogen) was used as secondary antibody.

Immunofluorescent staining

Cells were fixed with 4% PFA (Sangon Biotech) for 15 minutes, permeabilized with 0.1% Triton X (Sangon Biotech) for 5 minutes, and blocked with 3% BSA (Sigma-Aldrich) for 1 hour. Cells were subsequently stained with appropriate primary antibodies and AlexaFluor conjugated secondary antibodies (Life Technologies). Nuclei were stained with DAPI (Roche Diagnostics). For the staining of pluripotency markers, the primary antibodies were OCT4 (Santa Cruz Biotechnology), NANOG (Santa Cruz Biotechnology), SSEA-4 (Abcam) and SOX2 (Abcam). For the staining of cardiac-specific markers, the primary antibodies were TNNT2 (Abcam) and α-actinin (Abcam). Pictures were taken with 60× objective on confocal microscope (Nikon, A1) using NIS-Elements AR software (Nikon).

Cell viability assay

The iPSC-CMs were cultured in 96-well plate. Cell viability analyses were performed using CCK8-based *in vitro* cell proliferation and cytotoxicity assay kit (Beyotime) according to the manufacturer´s instructions. Cells were incubated in the presence of 10 μl CCK8 reagent per well for 2 hours. Absorbance at 450 nm was measured using an iMark^{TM} microplate reader (BioRad).

Transmission electrical microscopy

The iPSC-CMs were dissociated with 0.25% Tripsin-EDTA, scrapped into a 1.5-ml microcentrifuge tube and centrifuged, and then fixed with 2.5% ice-cold glutaraldehyde in 0.1 M phosphate buffer solution overnight at 4°C. The specimen was post-fixed with 1% OsO_4 in phosphate buffer solution and dehydrated by a series of ethyl-alcohol (30%, 50%, 70%, 80%, 90%, 95% and 100%) for 15-20 minutes at each step, then transferred to absolute acetone for 20 minutes. Next, the specimen was placed in the 1:1 mixture of absolute acetone and final spur resin mixture for 1 hour at room temperature, and transferred to 1:3 mixture of absolute acetone and final spur resin mixture for 3 hours, and then transferred to final spur resin mixture overnight. The specimen was placed in a 1.5-ml tube contained spur resin, heated at 70°C for more than 9 hours, and sectioned using a LEICA EM UC7 ultratome. The sections were then stained with uranyl acetate and alkaline lead citrate for 5-10 minutes. Pictures were taken using a transmission electron microscopy (Hitachi, Model H-7650).

Reactive oxygen species (ROS) assay

Cellular levels of ROS in iPSC-CMs were determined using a Reactive Oxygen

Species Assay Kit (Beyotime) according to the manufacturer's instructions. The fluorescence intensity was measured by CytoFLEX LX Flow Cytometer (Beckman Coulter).

Multi-electrode array (MEA)

For cell preparation of the experiments, a 20 μl droplet of coating solution (Matrigel, BD) was applied on the area of the electrodes of 1-well or 6-well MEA probes (60MEA200/30iR-Ti-gr or 60-6wellMEA200/30iR-Ti-tcr, Multi Channel Systems, Germany), which were incubated at 37°C in 5% CO_2 for at least 1 hour. The iPSC-CMs were then dissociated from the 6-well plates using TrypLE (Life technologies), then reseeded onto recording wells at a density of 1-1.5×10^5 cells in 20 μl bead of the cell suspension per well. After incubation with 5% CO_2 at 37°C for 2 hours to promote adhesion, each well was filled with culture media to a final volume (1-well was 1 ml and 6-well was 500 μl). Culture medium was changed after 48 hours, afterwards be sure to exchange medium every two or three days throughout 5-7 days of culturing period. Field potentials were recorded from spontaneously beating iPSC-CMs using the MEA2100 data acquisition system (Multi Channel Systems, Germany) with sampling at 10 kHz. All experiments were performed at 37°C and began after a 20-minute equilibration period. The measured beating period (BP), field potential duration (FPD) and field potential amplitude (FPA) were analyzed with Cardio 2D$^+$ software (Multi Channel Systems, Germany). FPD is inversely related to beating rate, therefore FPD is adjusted for the beating rate to yield FPDc. Steady-state parameters were averaged, and FPD was normalized to beat rate using the Fridericia's

correction formula: FPDc= FPD/(inter-spike interval $)^{1/3}$, where inter-spike interval (ISI) indicates the time interval (in seconds).

Ionic current recordings from iPSC-CMs

The iPSC-CMs were mechanically and enzymatically dissociated to obtain single cells, which were seeded on Matrigel-coated glass coverslips (Warner Instruments). Cells with spontaneous beatings were selected and action potentials were recorded using an EPC-10 patch clamp amplifier (HEKA). Continuous extracellular solution perfusion was achieved using a rapid solution exchanger (Bio-logic Science Instruments). Data were acquired using PatchMaster software (HEKA) and digitized at 1 kHz. Data analyses were performed using Igor Pro (Wavemetrics) and Prism (Graphpad). A TC-344B heating system (Warner Instruments) was used to maintain the temperature at 35.5-37°C. Different ionic currents were recorded from single iPSC-CMs using the ruptured patch clamp technique with conventional voltage clamp protocols. For sodium current recordings, bath solution contained: 50 mM NaCl, 110 mM CsCl, 1.8 mM $CaCl_2$, 1 mM $MgCl_2$, 10 mM glucose, 10 mM HEPES and 0.001 mM Nifedipine (pH 7.4 with CsOH). Pipette solutions contained: 10 mM NaCl, 135 mM CsCl, 2 mM $CaCl_2$, 5 mM MgATP, 5 mM EGTA, and 10 mM HEPES (pH 7.2 with CsOH). For calcium current recordings, bath solution contained: 160 mM TEA-Cl, 5 mM $CaCl_2$, 1 mM $MgCl_2$, 10 mM glucose, 10 mM HEPES, 0.01 mM TTX, 2 mM 4-AP (pH 7.4 with CsOH). Pipette solutions contained: 145 mM CsCl, 5 mM NaCl, 1 mM $CaCl_2$, 5 mM MgATP, 5 mM EGTA, and 10 mM HEPES (pH 7.2 with CsOH). For total potassium current recordings, bath solution contained: 150 mM

NaCl, 5.4 mM KCl, 1.8 mM $CaCl_2$, 1 mM $MgCl_2$, 15 mM Glucose, 15 mM HEPES, 1 mM Na-Pyruvate and 0.002 mM Nifedipine (pH 7.4 with NaOH). Pipette solutions contained: 150 mM KCl, 5 mM NaCl, 2 mM $CaCl_2$, 5 mM EGTA, 10 mM HEPES and 5 mM Mg-ATP (pH 7.2 with KOH). All currents were normalized to cell capacitance to obtain current density. Steady-state activation and inactivation curves were fitted by using a Boltzmann equation: $f = 1/\{1+ \exp [\pm(V - V_{1/2})/k]\}$, in which $V_{1/2}$ is half-maximum (in)activation potential and k is slope factor.

I_{Kr} recordings in transfected human embryonic kidney 293 cells by patch clamp

Human embryonic kidney 293 cells (HEK293 cells) were transiently transfected with 0.5 μg of WT KCNH2 plasmids using Lipofectamine 3000 (Invitrogen) according to the manufacturer's instructions. Bath solution contained: 140mM NaCl, 5 mM KCl, 1 mM $MgCl_2$, 2 mM $CaCl_2$, 10 mM HEPES and 10 mM glucose (pH 7.4 with NaOH). Pipette solutions contained: 140 mM KCl, 10 mM NaCl, 1 mM $MgCl_2$, 10 mM HEPES and 10 mM EGTA (pH 7.2 with KOH).

RNA-Sequencing

Total RNA of iPSC-CMs was extracted using RNeasy Mini Kit (Qiagen). The mRNA was purified from total RNA using poly-T oligo-attached magnetic beads. The prepared sequencing library were sequenced on an Illumina Novaseq platform and 150 bp paired-end reads were generated. The sequencing throughput was > 4 Gb. The reads have been submitted to NCBI SRA database under the accession number PRJNA730982. The raw reads were processed by Trimmomatic v0.35 for quality control. The obtained reads were aligned to the human reference genome using Hisat2

v2.0.5. Differential expression analysis was performed using the DESeq2 R package v1.20.0. Gene Ontology (GO) and KEGG enrichment analysis of differentially expressing genes was implemented by the clusterProfiler R package. The enriched GO terms and KEGG pathways were visualized by the REVIGO online service tool and the pathview R package, respectively.

Western blot

The iPSC-CMs were grown in 6-well plates to 80% confluence, detached with TrypLE, and then pelleted at 12000 rpm for 3-5 minutes at 4°C. After washing with DPBS, the pellets were re-suspended in 50-100 µl lysis buffer. Lysates were placed on ice for 30 minutes and the supernatants were collected after centrifuging at 12000 rpm for 5 minutes. Protein concentration was measured using a BCA kit (Pierce). Western blots were performed with the following antibodies: LC3 (Sigma), SQSTM1/p62 (MBL), Beclin 1 (Abcam), LAMP-2 (Santa Cruz Biotechnology), Cathespin D (Cell Signaling Technology) and GAPDH (MultiSciences Biotech).

Live-Cell confocal microscopy

The iPSC-CMs were grown in a confocal dish (NEST) and treated with LysoTracker probes (Invitrogen/Molecular Probes) to label lysosomes. The probe stock was diluted to a final working concentration of 50 nM with RPMI 1640 medium (without phenol red) (Gibco). Before start, the culture medium was aspirated from cells grown on dishes, the cells were then washed three times using RPMI 1640 medium before and after incubating with probe-containing medium for 2 hours under the desired growth

conditions. The living cells were observed on confocal microscope (Leica, DMI8) and pictures were taken with 63× objective.

mCherry-GFP-LC3 plasmid transfection

The mCherry-GFP-LC3 plasmid was obtained as a gift from Dr. Qiming Sun. The iPSC-CMs prepared in a confocal dish were transfected with this plasmid using Lipofectamine^{TM} 3000 (Invitrogen) following the manufacturer's instructions. Pictures were taken with 63× objective using confocal microscope (Leica, DMI8) following the live-cell confocal microscopy.

Compounds and solutions

All the chemicals used in the electrophysiological experiments were purchased from Sigma-Aldrich. Azithromycin was purchased from Tocris and stock solutions were prepared in 30 mM in DMSO. Moxifloxacin was purchased from Selleck and stock solutions were prepared in 50 mM in DMSO. Catechin hydrate was purchased from Beyotime and stock solutions were prepared in 50 mg/ml in DMSO. Torin was purchased from Selleck and stock solutions were prepared in 10 μM in DMSO. MRT68921 was purchased from Selleck and stock solutions were prepared in 1 mM in DMSO. Bafilomycin A1 was purchased from Selleck and stock solutions were prepared in 50 μM in DMSO. Chloroquine was purchased from Sigma and stock solutions were prepared in 20 mM in DMSO.

Statistical analysis

Statistical significance was determined by unpaired two-tailed Student's t-test to compare two groups and by One-way ANOVA followed by Tukey post hoc test to

compare multiple groups. All data have passed normality testing by Kolmogorov-Smirnov test. A p value of < 0.05 was considered statistically significant. Data were shown as mean ± sem and analyzed by GraphPad Prism 6 (GraphPad Software).

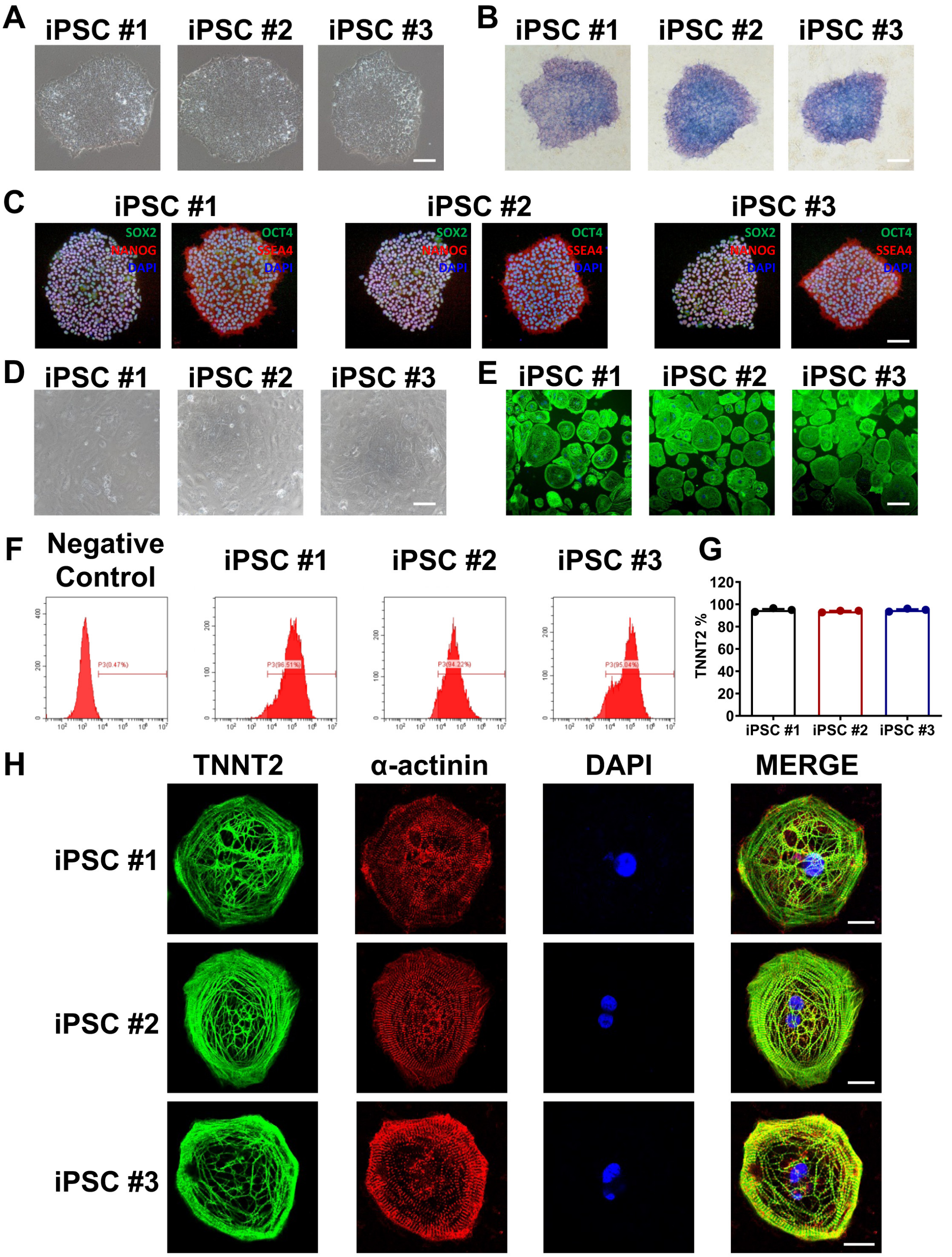

Figure S1. Generation and characterization of induced pluripotent stem cell-derived cardiomyocytes (iPSC-CMs). A. Typical morphology of iPSCs derived from three healthy control subjects (iPSC #1, iPSC #2 and iPSC #3). Scale bar = 100 µm. **B.** Alkaline phosphatase (ALP) staining of three iPSC lines. Scale bar = 100 µm. **C.** Pluripotent staining of three iPSC lines using SOX2 (green), NANOG (red), OCT4 (green) and SSEA4 (red). DAPI indicates nuclear staining (blue). Scale bar = 100 µm.

D. Representative brightfield imaging of three iPSC-CM lines. Scale bar = 100 µm. **E.** Representative graphs of TNNT2 staining of three iPSC-CM lines. Scale bar = 100 µm. **F.** Fluorescence-activated cell sorting (FACS) analysis of TNNT2-positive cells in three iPSC-CM lines. **G.** Bar graph to compare the percentage of TNNT2-positive cells between three iPSC-CM lines. n= 3. A 2D *in vitro* monolayer protocol was employed to differentiate iPSCs into cardiomyocytes**,** which can give rise to a yield of > 90% after purification evidenced by FACS analysis. **H.** Immunofluorescent staining of three iPSC-CM lines using cardiac-specific markers TNNT2 (green) and α-actinin (red). DAPI indicates nuclear staining (blue). Scale bar = 20 µm.

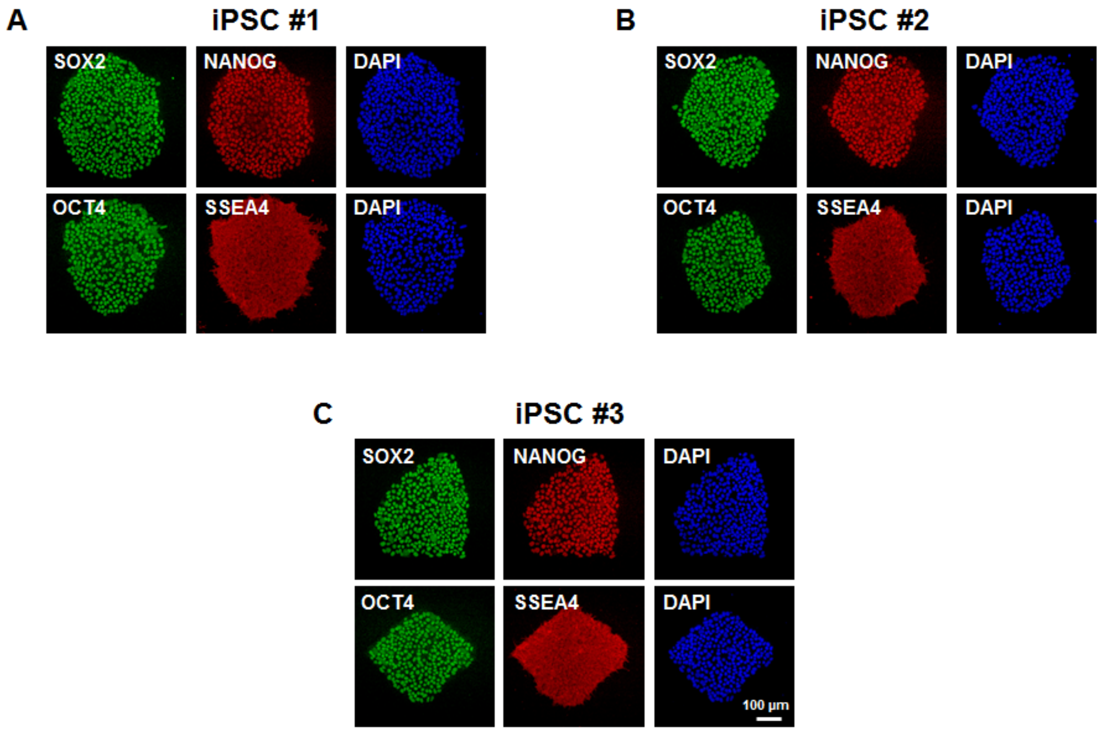

Figure S2. Pluripotent staining of iPSCs. A-C. Pluripotent staining of three iPSC lines (iPSC #1, iPSC #2 and iPSC #3) using SOX2 (green), NANOG (red), OCT4 (green) and SSEA4 (red). DAPI indicates nuclear staining (blue). Scale bar = 100 µm.

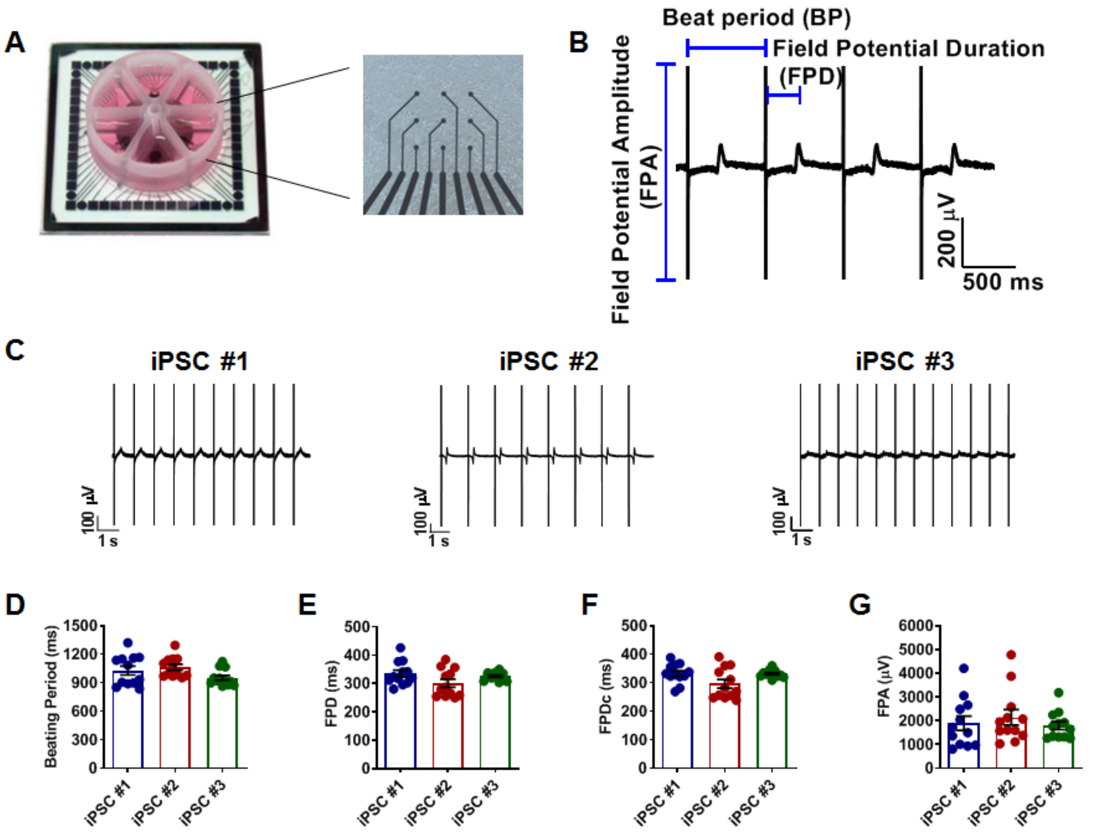

Figure S3. Baseline multi-electrode array (MEA) electrophysiology. A. Representative image of monolayer iPSC-CMs plated on a 6-well MEA probe. **B.** Schematic diagram of a MEA trace showing key field potential parameters to analyze. **C.** Representative field potential tracings recorded from three iPSC-CM lines at baseline by MEA. **D-G.** Bar graphs to compare beating period (BP), filed potential duration (FPD), corrected FPD (FPDc) and filed potential amplitude (FPA) at baseline between three iPSC-CM lines. n= 12. Baseline electrophysiological parameters were comparable between three iPSC-CM lines, including beating period (average value: 952.7-1062.0 ms), FPD (average value: 301.2-334.9 ms), corrected FPD (FPDc) (average value: 296.5-332.5 ms) and FPA (average value: 1793.0-2141.0 µV).

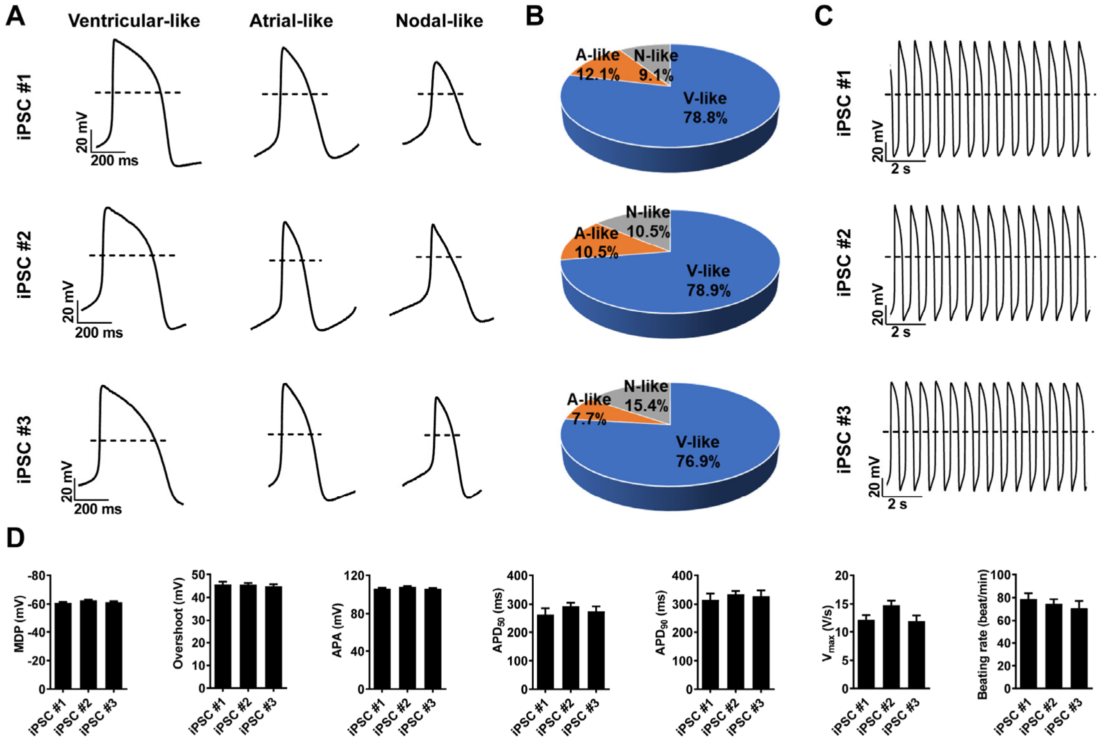

Figure S4. Action potential recordings of three different iPSC-CM lines by single-cell patch clamp. A. Representative action potential tracings of ventricular-, atrial- and nodel-like myocytes derived from iPSC #1, iPSC #2 and iPSC #3. Dash lines indicate 0 mV. **B.** Pie graphs to show the proportion of ventricular- (V-like), atrial- (A-like) and nodel-like (N-like) myocytes in three different iPSC-CM lines. **C.** Representative action potential waveforms recorded from ventricular-like myocytes derived from three different iPSC lines. **D.** Bar graphs to compare key parameters of action potentials between three different iPSC-CM lines, including maximal diastolic potential (MDP), overshoot, action potential amplitude (APA), AP duration at 50% repolarization (APD_{50}), AP duration at 90% repolarization (APD_{90}), maximal upstroke velocity (V_{max}) and beating rate.

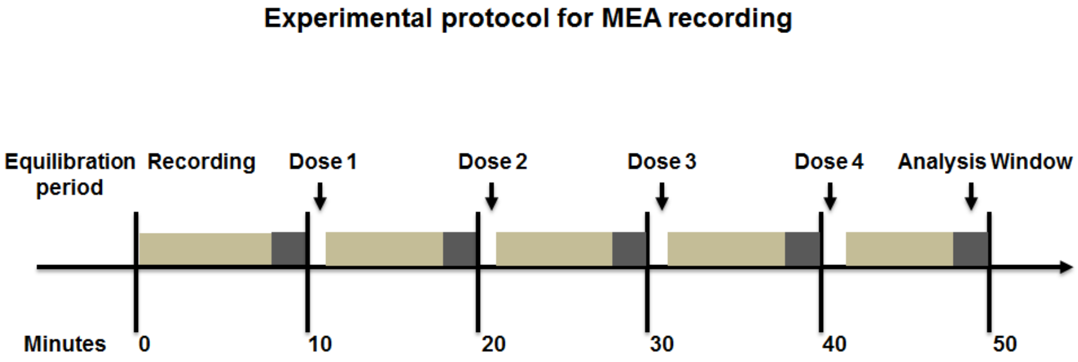

Figure S5. Schematic demonstration of experimental protocol for MEA recordings to characterize electrophysiological effects of acute AZM in iPSC-CMs.

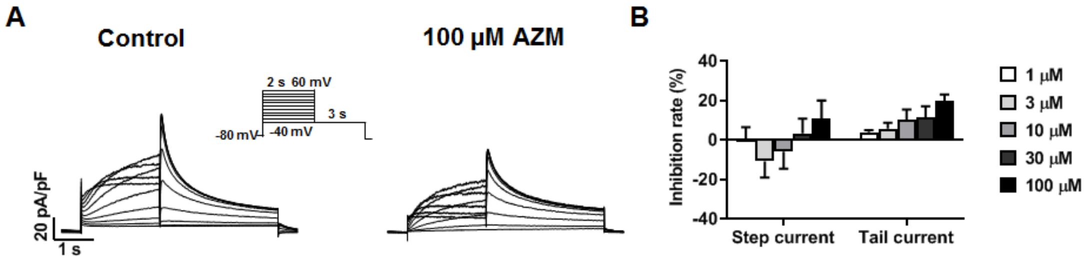

Figure S6. Effects of acute AZM on hERG currents recorded from HEK293 cells. **A.** Representative hERG current (I_{Kr}) tracings recorded from HEK293 cells at baseline and acute treatment of 100 μM AZM. **B.** Bar graph to compare the inhibition rate of hERG currents by acute treatment of AZM at different concentrations. n= 4-5.

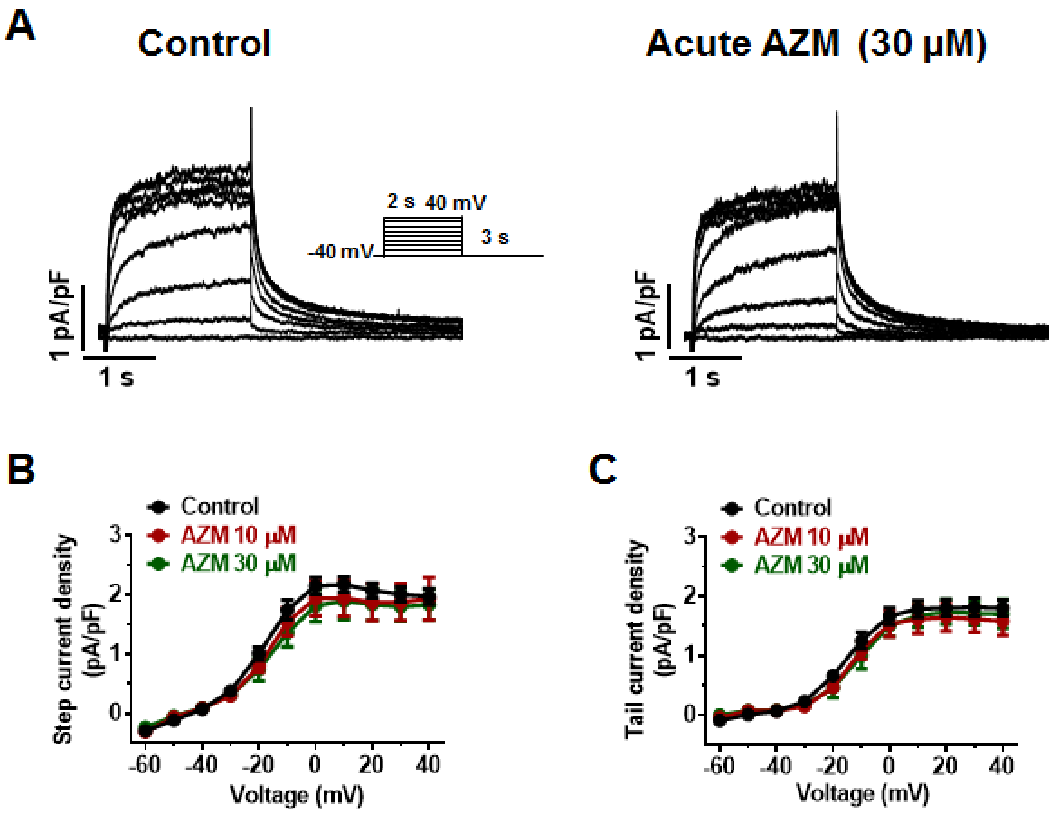

Figure S7. Effects of acute AZM on total K^+ currents in iPSC-CMs. A. Representative total K^+ current tracings isolated from control iPSC-CMs and iPSC-CMs with acute treatment of 30 µM AZM. **B-C.** Comparison of K^+ current-voltage relationship curves (IV curves) between control iPSC-CMs and iPSC-CMs with acute treatment of 10 and 30 µM AZM. Myocytes were derived from iPSC #3. n= 3-8.

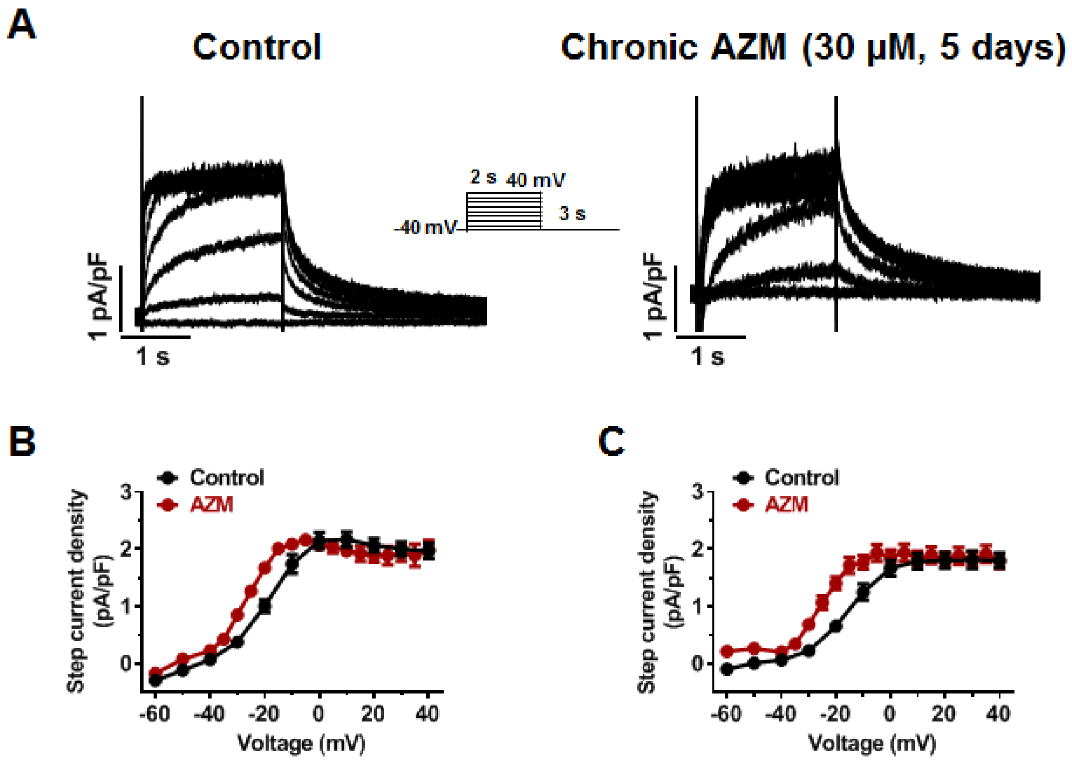

Figure S8. Effects of chronic AZM on total K^+ currents in iPSC-CMs. A. Representative total K^+ current tracings isolated from control iPSC-CMs and iPSC-CMs with chronic treatment of AZM (30 µM, 5 days). **B-C.** Comparison of total K^+ IV curves between control iPSC-CMs and iPSC-CMs with chronic treatment of AZM (30 µM, 5 days). Myocytes were derived from iPSC #3. n= 5-22.

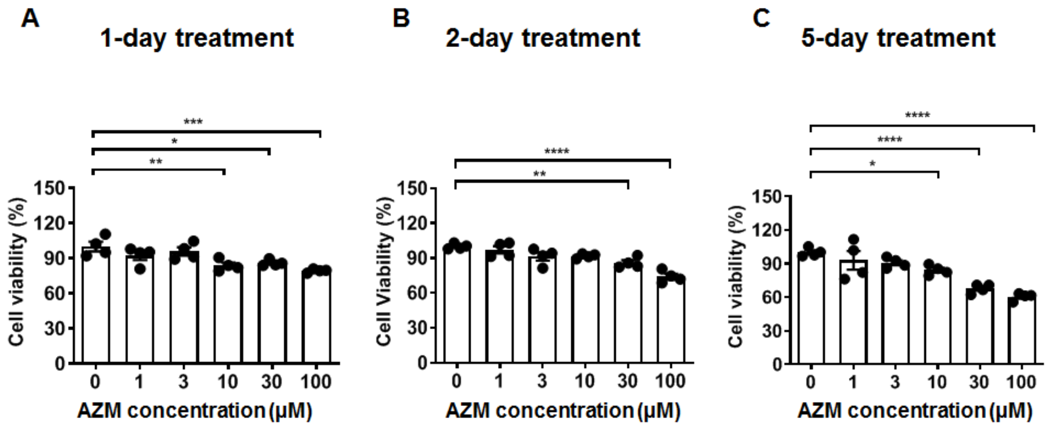

Figure S9. Effects of chronic AZM on the cell viability of iPSC-CMs in concentration- and time-dependent manners. A-C. Bar graphs to compare the cell viability between control and AZM-treated iPSC-CMs in concentration-dependent (0, 0.1, 0.3, 1, 3, 10, 30, 60 and 100 μM) and time-dependent (1-day, 2-day and 5-day) manners. Myocytes were derived from iPSC #3. n= 4. $^{*}P< 0.05$, $^{**}P< 0.01$, $^{***}P< 0.001$ and $^{****}P< 0.0001$.

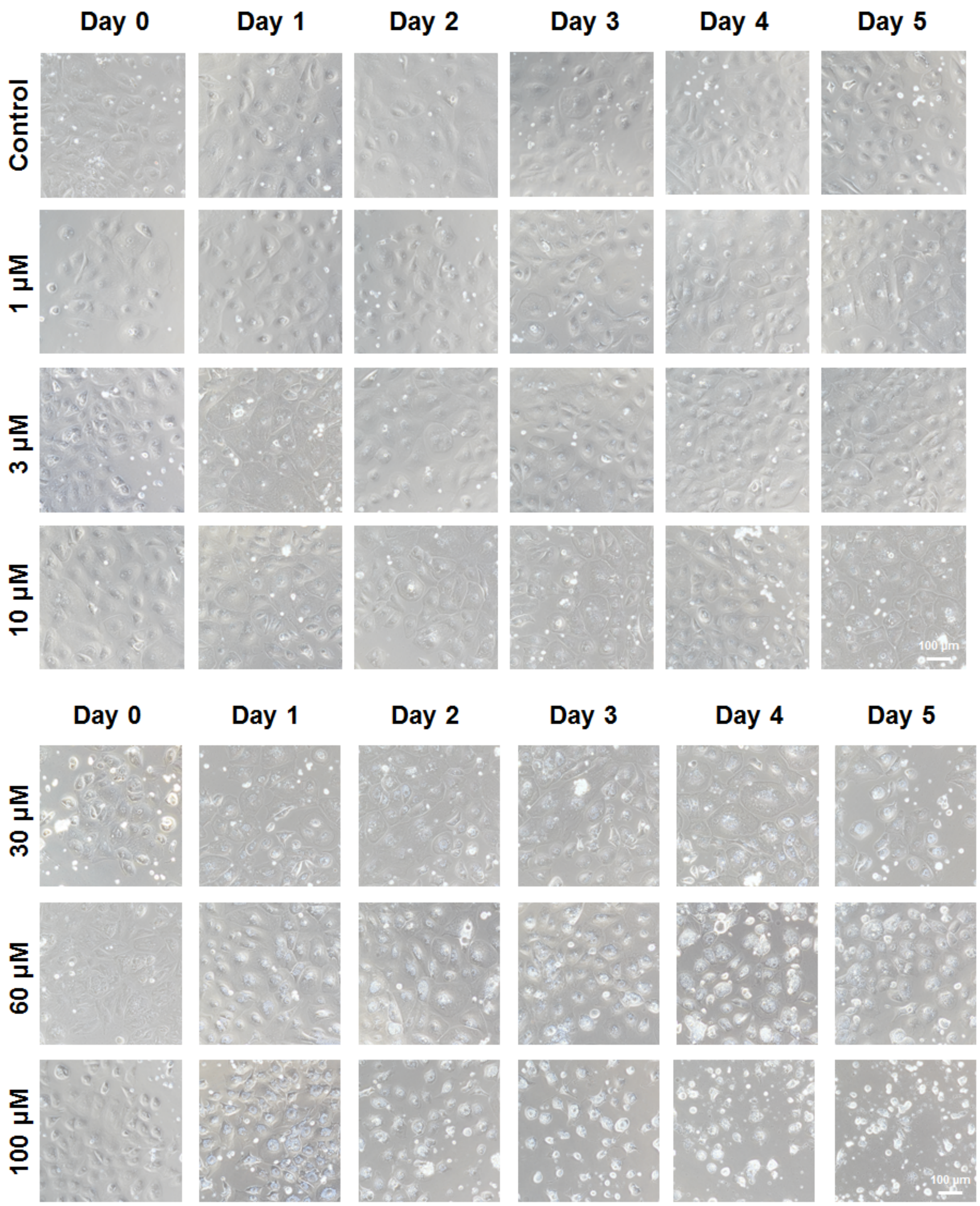

Figure S10 and S11. Effects of chronic AZM on the morphology of iPSC-CMs in concentration- and time-dependent manners. Representative live-cell images of AZM-treated iPSC-CMs in concentration-dependent (0, 0.1, 0.3, 1, 3, 10, 30, 60 and 100 µM) and time-dependent (1-day, 2-day, 3-day, 4-day and 5-day) manners. Myocytes were derived from iPSC #3. Scale bar = 100 µm. Intracellular vacuoles were observed in AZM-treated iPSC-CMs started from 30 µM on day 1 postinduction and the phenotype became exacerbated following the 5-day AZM treatment.

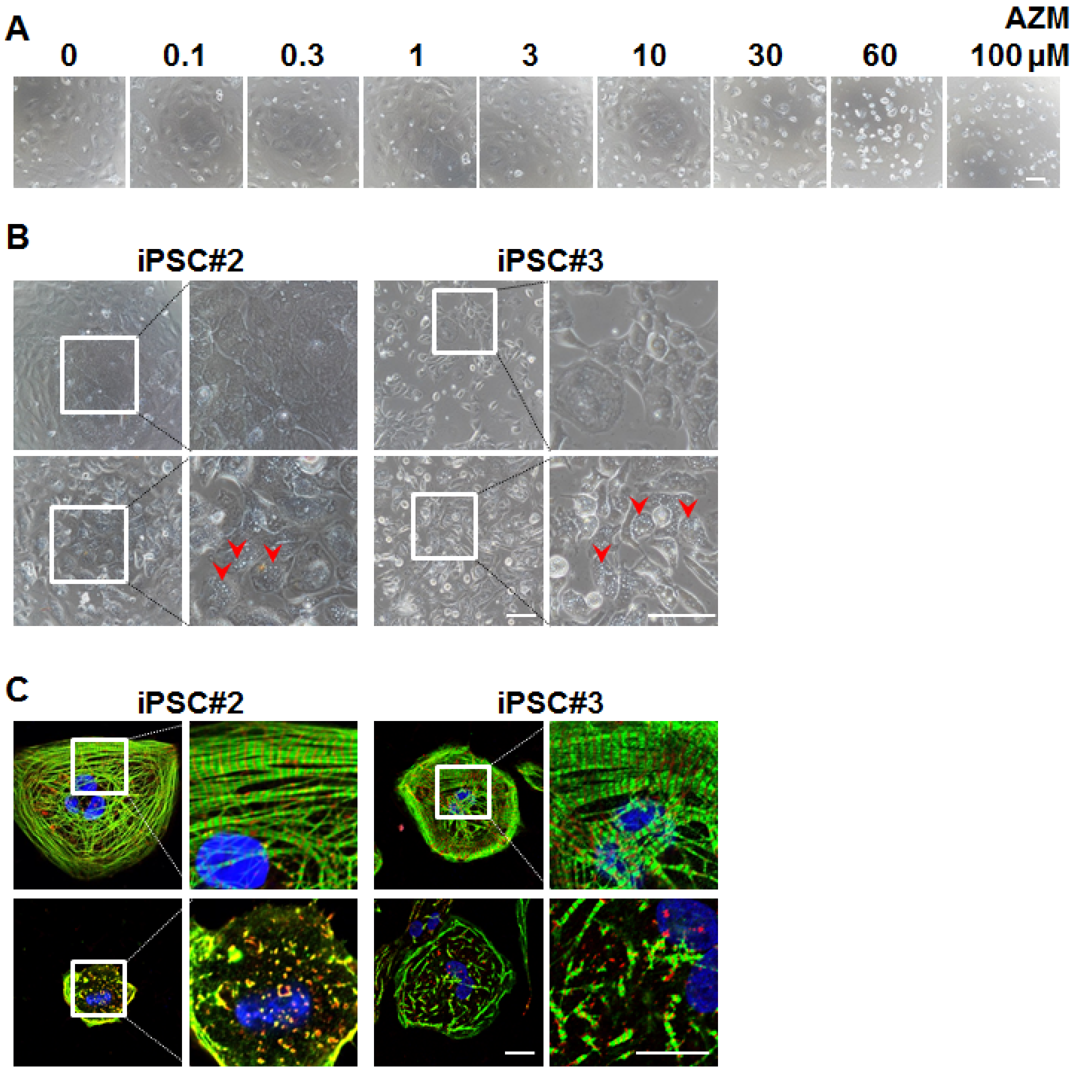

Figure S12. AZM-induced morphological changes in iPSC-CMs. A. Representative live-cell images of iPSC-CMs treated with 0, 0.1, 0.3, 1, 3, 10, 30, 60 and 100 µM AZM. Myocytes were derived from iPSC #3. Scale bar = 100 µm. **B.** Representative morphology of control and 30 µM AZM-treated myocytes derived from iPSC #2 and iPSC #3. Red arrows indicate the vacuole formation. Scale bar = 100 µm. **C.** Immunofluorescent staining of control and 30 µM AZM-treated myocytes derived from iPSC #2 and iPSC #3 using TNNT2 (green) and α-actinin (red). DAPI indicates nuclear staining (blue). Enlarged views showing cardiac sarcomeres in the two groups. Scale bar = 20 µm.

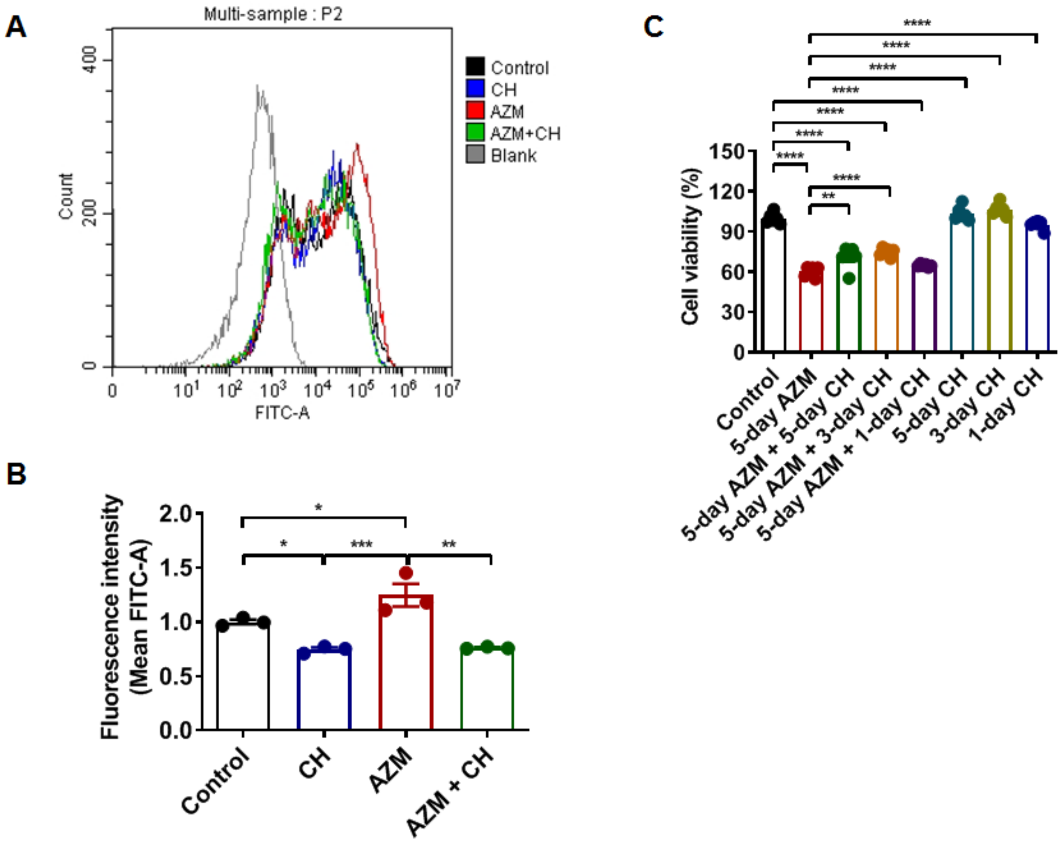

Figure S13. Elevated reactive oxygen species (ROS) in AZM-treated iPSC-CMs. **A.** Measurement of cellular ROS amount by flow cytometry in control iPSC-CMs, AZM-treated iPSC-CMs, and iPSC-CMs treated with AZM and Catechin Hydrate (CH) (40 µg/ml, 3 days) as an anti-oxidant. Myocytes were derived from iPSC #3. **B.** Bar graph to compare the cellular ROS amount between different groups in a. n= 3. $^{*}P< 0.05$, $^{**}P< 0.01$ and $^{***}P< 0.001$. **C.** Bar graph to compare the cell viability between control and AZM-treated myocytes derived from iPSC #3 with time-course CH application. n= 6. $^{**}P< 0.01$ and $^{****}P< 0.0001$.

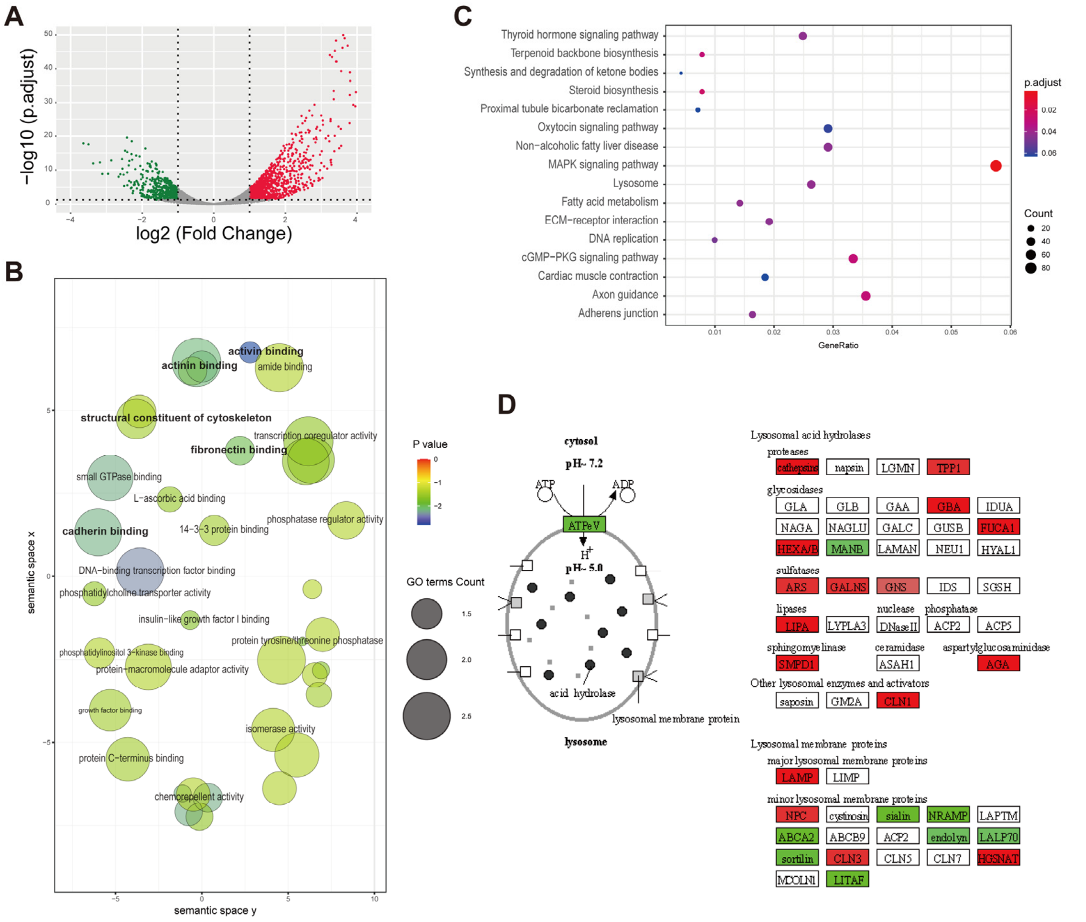

Figure S14. Comparison of transcriptome between control and AZM-treated iPSC-CMs. A. Volcano plot showing the differentially expressing genes (DEGs). Red dots, genes up-regulated in AZM-treated iPSC-CMs; green dots, genes up-regulated in control iPSC-CMs; grey dots, genes not differentially expressed between the two groups. A total of 3273 (27.3%) genes showed varied expression, with 1413 being up-regulated and 1860 being down-regulated in the AZM-treated iPSC-CMs. This large number of DEGs was in line with the dramatic morphologic change of the AZM-treated iPSC-CMs. **B.** Semantic space analysis of significantly enriched Gene Ontology (GO) terms for the DEGs. Bubble color indicates the extent for which GO terms were enriched, and bubble size indicates the frequency of the GO terms. Based on GO annotation, a number of DEGs were associated with structural constituent of cytoskeleton, as well as the binding of actinin, actin, α-actinin, fibronectin, and cadherin. **C.** Bubble diagram of Kyoto Encyclopedia of Genes and Genomes (KEGG) enrichment analysis. Gene ratio indicates the number of the genes enriched in the

pathway divided by the number of all genes in background gene set. Based on KEGG annotation, the DEGs were enriched in 16 pathways, including adherens junction (hsa04520), ECM-receptor interaction (hsa04512), cardiac muscle contraction (hsa04260) and lysosome (hsa04142). **D.** Illustration of the KEGG pathway lysosome (hsa04142). The up- and down-regulated DEGs are marked in red and green, respectively. Notably, majority of the lysosomal acid hydrolases were upregulated in AZM-treated iPSC-CMs, whereas the lysosomal membrane proteins could be either upregulated or downregulated.

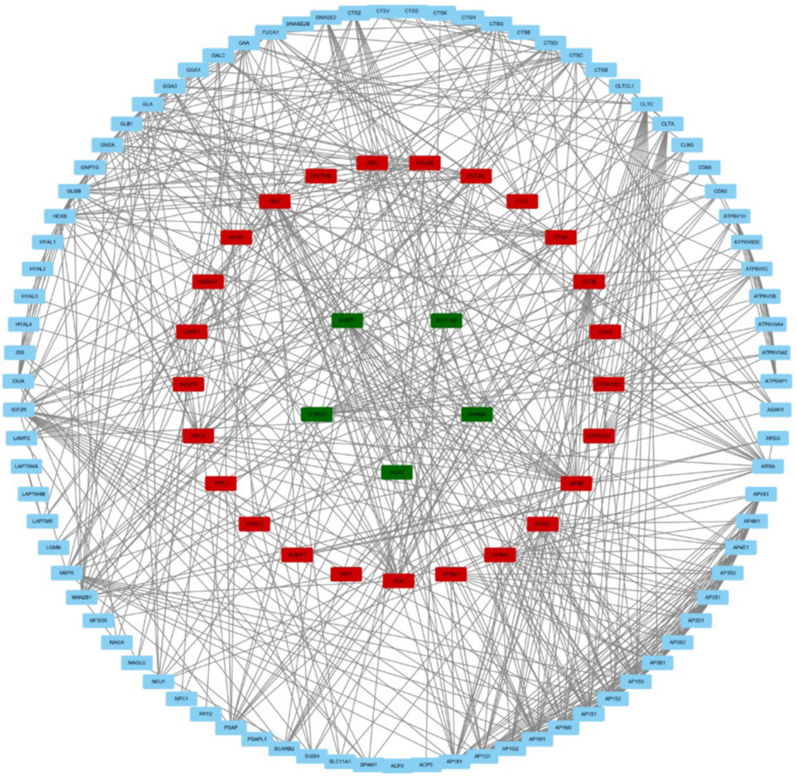

Figure S15. Protein-protein interaction of genes involved in the KEGG pathway lysosome (hsa04142). The red and green genes represent the up- and down-regulated in AZM-treated iPSC-CMs, respectively; grey genes are not differentially expressed between the two groups. The analysis of protein-protein interaction was performed using the online tool of the STRING database (https://string-db.org/).

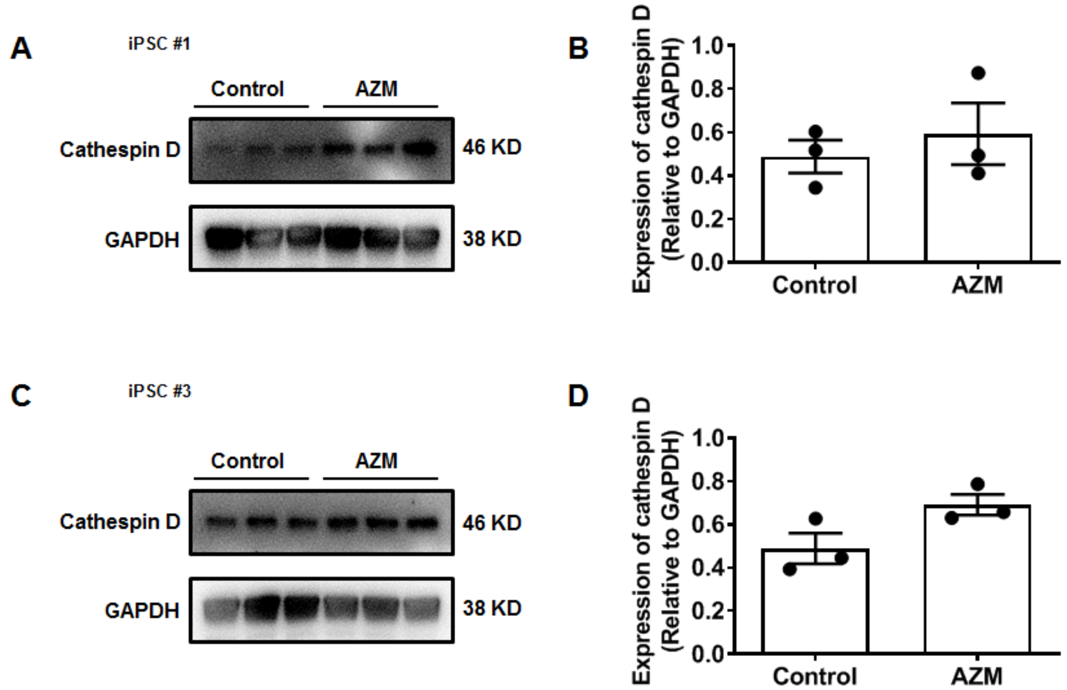

Figure S16. Comparison of protein expression of cathepsin D between control and AZM-treated iPSC-CMs. A & C. Western blot analysis of the cathepsin D expression in control and AZM-treated myocytes derived from iPSC #1 and iPSC #3, respectively. **B & D.** Bar graph to compare the cathepsin D expression between control and AZM-treated myocytes derived from iPSC #1 and iPSC #3, respectively. n= 3.

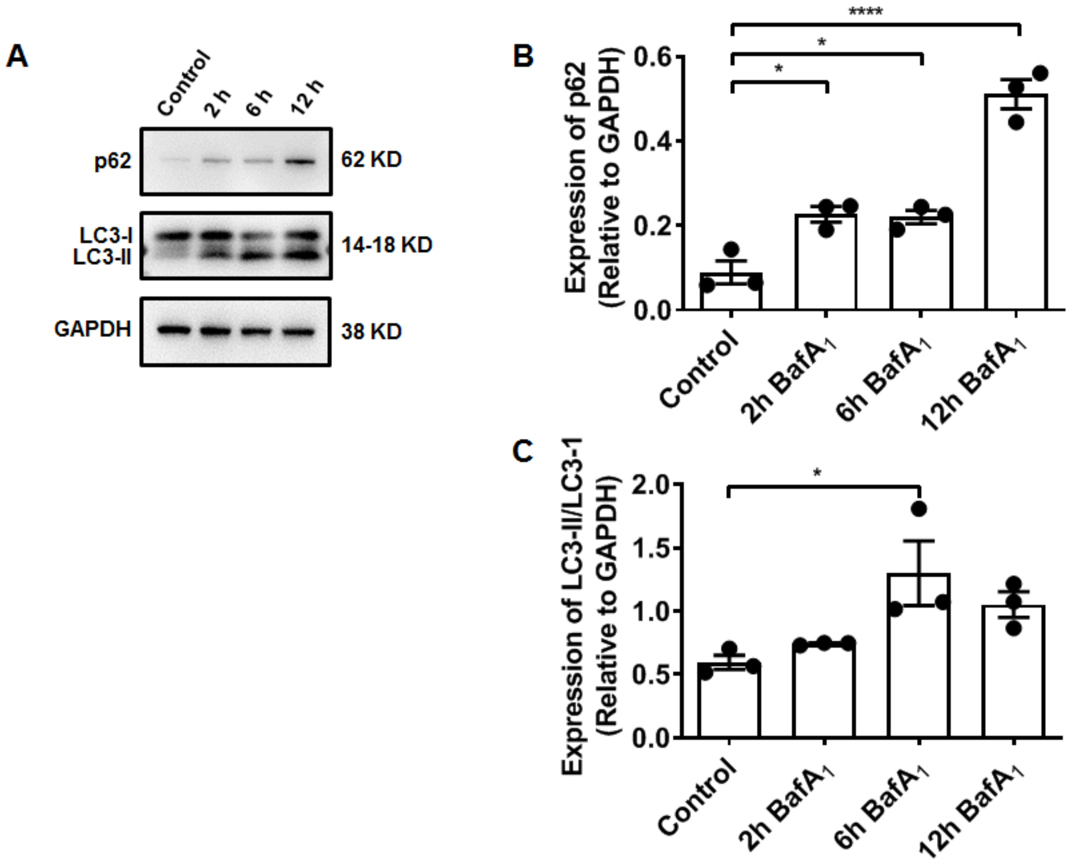

Figure S17. Effects of BafA$_1$ treatment on p62 and LC3-II/LC3-I expression at the protein level in iPSC-CMs. A. Western blot analysis of the p62 and LC3-II/LC3-I expression in control and BafA$_1$-treated myocytes derived from iPSC #3 for 2, 6 and 12 hours, respectively. **B-C.** Bar graphs to compare the p62 and LC3-II/LC3-I expression between control and BafA$_1$-treated iPSC-CMs. n= 3. $^{*}P< 0.05$ and $^{****}P< 0.0001$.

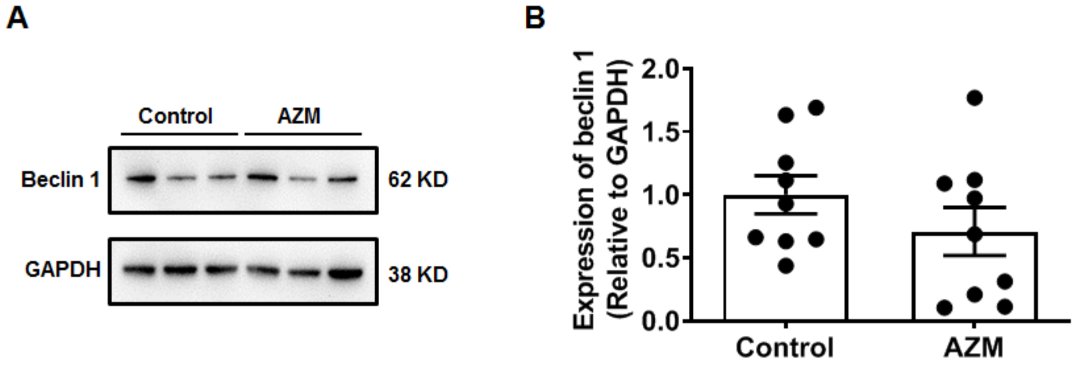

Figure S18. Comparison of protein expression of beclin 1 between control and AZM-treated iPSC-CMs. A. Western blot analysis of the beclin 1 expression in control and AZM-treated myocytes derived from iPSC #1. **B.** Bar graph to compare the beclin 1 expression between control and AZM-treated iPSC-CMs. n= 9.

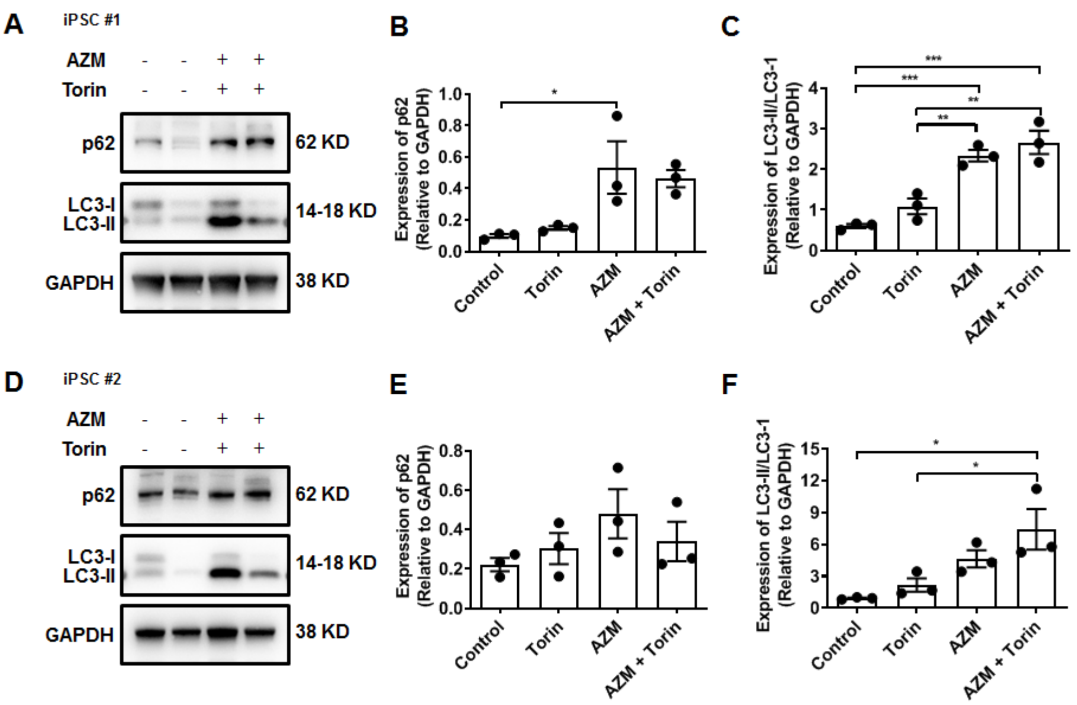

Figure S19. Effects of Torin on elevated p62 and LC3-II/LC3-I expression in AZM-treated iPSC-CMs. A & D Western blot analysis of p62 and LC3-II/LC3-I expression in control and AZM-treated myocytes derived from iPSC #1 and iPSC #2 with or without Torin (2.5 nM, 5 days). **B-C & E-F** Bar graphs to compare p62 and LC3-II/LC3-I expression between different groups in A & D. n= 3. $^{*}P< 0.05$, $^{**}P< 0.01$ and $^{***}P< 0.001$.

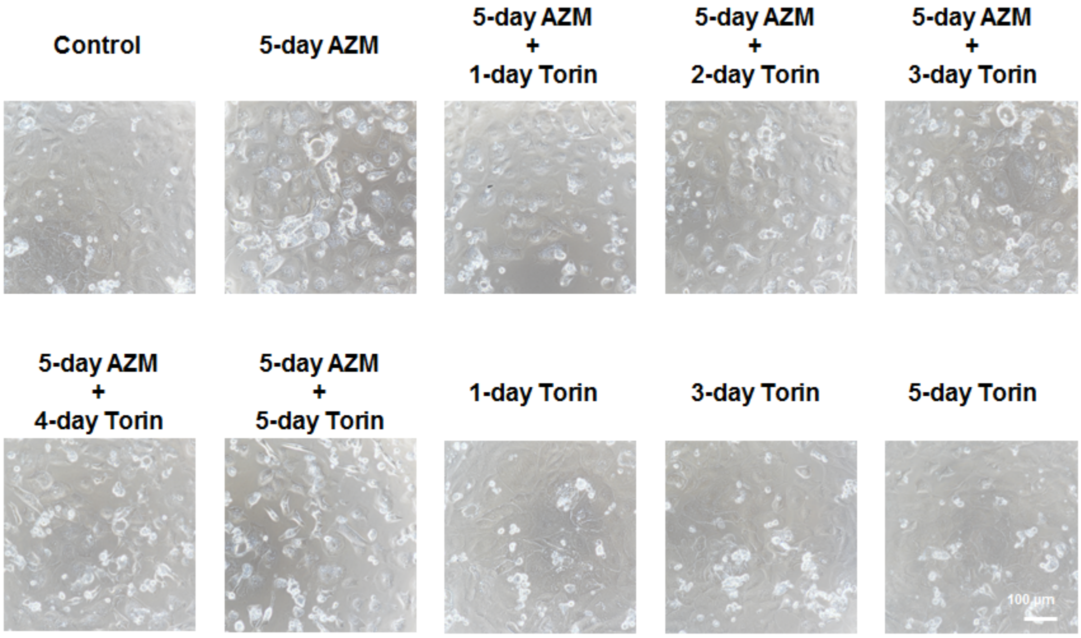

Figure S20. Effects of Torin on cell viability in AZM-treated iPSC-CMs. Representative live-cell images of control and AZM-treated myocytes derived from iPSC #1 with time-course Torin application. Scale bar = 100 µm.

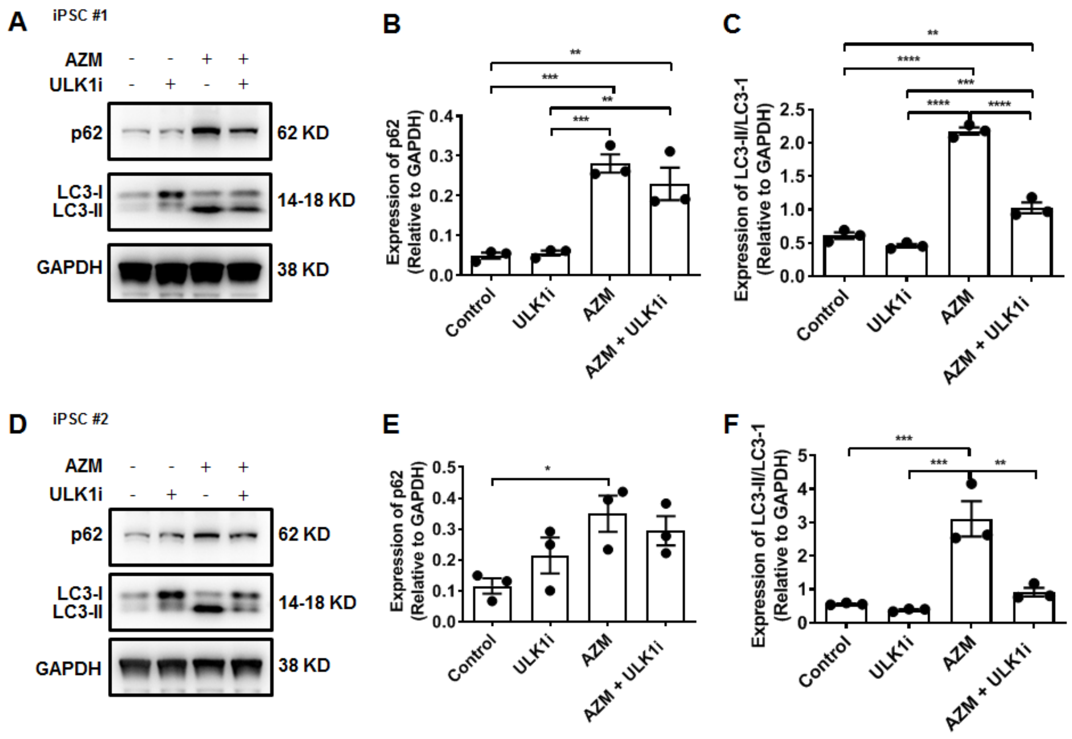

Figure S21. Effects of ULK1 inhibitor on elevated p62 and LC3-II/LC3-I expression in AZM-treated iPSC-CMs. A and D. Western blot analysis of p62 and LC3-II/LC3-I expression in control and AZM-treated myocytes derived from iPSC #1 and iPSC #2 with or without ULK1 inhibitor (ULK1i) MRT68921 (1 µM, 5 days). **B-C & E-F.** Bar graphs to compare p62 and LC3-II/LC3-I expression between different groups in A & D. n= 3. $^{*}P< 0.05$, $^{**}P< 0.01$, $^{***}P< 0.001$ and $^{****}P< 0.0001$.

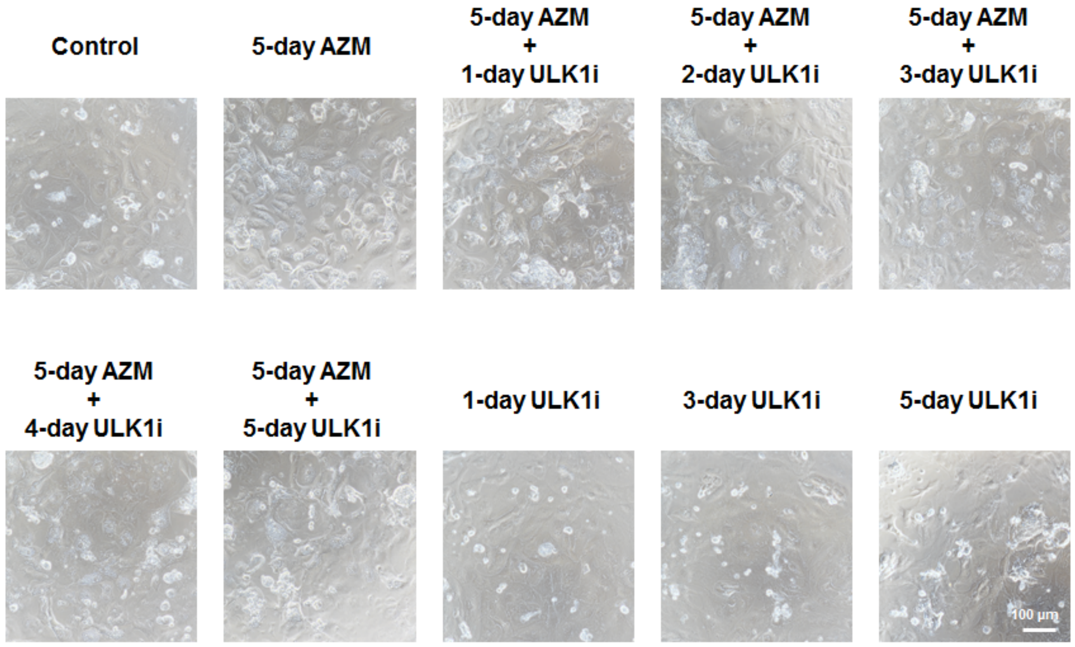

Figure S22. Effects of ULK1i on cell viability in AZM-treated iPSC-CMs. Representative live-cell images of control and AZM-treated myocytes derived from iPSC #1 with time-course ULK1i application. Scale bar = 100 µm.

Table S1. Acute effects of AZM or MXF treatment on FP parameters in iPSC-CMs

Drugs	Concentrations	Beat period (% of change)	FPD (% of change)	FPDc (% of change)	FPA (% of change)
**DMSO**	0.1%	-6.0 ± 6.2	-0.08 ± 1.5	2.2 ± 1.4	0.8 ± 8.9
	0.3%	-4.2 ± 2.4	-5.2 ± 4.6	-3.8 ± 4.3	-11.2 ± 3.4
	0.5%	-2.0 ± 3.5	-3.2 ± 5.6	-2.5 ± 5.6	-6.1 ± 4.6
**AZM**	1 μM	-9.4 ± 2.3	-11.0 ± 2.5	-8.1 ± 2.0	-1.7 ± 3.6
	3 μM	-26.6 ± 4.2	-35.0 ± 4.0	-28.0 ± 3.7	-21.5 ± 7.7
	10 μM	-51.0 ± 5.3	-60.4 ± 3.0	-49.3 ± 3.2	-32.7 ± 5.3
	30 μM	-64.6 ± 0.6	-70.6 ± 1.9	-58.5 ± 2.5	-60.8 ± 7.0
**MXF**	3 μM	-0.5 ± 0.7	7.1 ± 1.3	7.3 ± 1.4	-3.0 ± 4.5
	10 μM	-4.1 ± 2.3	13.0 ± 2.1	14.8 ± 2.5	-6.6 ± 9.9
	30 μM	15.4 ± 1.2	51.7 ± 5.3	42.4 ± 4.8	-49.4 ± 8.8
	100 μM	19.3 ± 7.8	104.5 ± 16.0	92.8 ± 12.7	-59.7 ± 17.6

Table S2. Acute effects of AZM treatment on Ca^{2+} and Na^{+} currents in iPSC-CMs

Ionic currents	Concentrations (μM)	Peak current density (pA/pF)	Cell capacitance (pF)	Steady-state activation		Steady-state inactivation	
				$V_{1/2}$ (mV)	k	$V_{1/2}$ (mV)	k
Ca^{2+} currents	0	-29.8 ± 1.8	58.1 ± 3.6	-15.8 ± 0.9	3.6 ± 0.2	-27.7 ± 0.8	5.1 ± 0.1
At 0 mV	1	-25.5 ± 2.1	60.4 ± 3.3	-13.3 ± 0.9	3.9 ± 0.3	-26.5 ± 0.6	4.9 ± 0.1
(n= 8-13)	10	-20.4 ± 1.4	58.0 ± 3.8	-14.5 ± 1.0	4.1 ± 0.4	-27.3 ± 0.6	5.0 ± 0.1
	30	-14.3 ± 1.0	58.1 ± 3.9	-11.6 ± 0.9	4.8 ± 0.3	-28.5 ± 0.6	5.5 ± 0.2
Na^{+} currents	0	-107.9 ± 8.0	28.2 ± 2.0	-37.9 ± 1.2	2.8 ± 0.4	-69.6 ± 0.8	6.1 ± 0.2
At -30 mV	10	-103.1 ± 8.6	27.3 ± 2.5	-41.5 ± 1.7	2.4 ± 0.4	-72.5 ± 1.2	5.7 ± 0.2
(n= 5-11)	30	-49.8 ± 6.2	30.7 ± 4.3	-37.3 ± 2.0	3.3 ± 0.5	-74.8 ± 1.5	5.9 ± 0.5

Table S3. Chronic effects of AZM treatment on FP parameters in iPSC-CMs

Drugs	Days postinduction	Beat period (% of change)	FPD (% of change)	FPDc (% of change)	FPA (% of change)
**0.1% DMSO**	Day 1	-14.5 ± 1.5	-10.5 ± 3.2	-5.6 ± 3.2	5.7 ± 4.3
	Day 2	-14.6 ± 2.4	-8.2 ± 2.7	-3.9 ± 2.4	-2.0 ± 6.3
	Day 3	-11.0 ± 2.2	-8.8 ± 3.4	-5.1 ± 3.8	7.7 ± 2.6
	Day 4	-2.2 ± 2.9	-11.2 ± 2.6	-10.4 ± 3.0	6.5 ± 2.2
	Day 5	-5.5 ± 3.5	-6.9 ± 2.7	-5.0 ± 2.6	2.5 ± 4.0
**30 µM AZM**	Day 1	-42.6 ± 2.8	-63.8 ± 2.5	-56.7 ± 2.5	135.8 ± 19.9
	Day 2	-35.1 ± 2.6	-56.3 ± 2.4	-49.8 ± 2.5	153.0 ± 36.3
	Day 3	-36.9 ± 2.3	-61.1 ± 1.6	-54.7 ± 1.6	193.4 ± 30.0
	Day 4	-28.0 ± 4.2	-54.2 ± 2.3	-49.0 ± 2.2	127.2 ± 33.5
	Day 5	-30.6 ± 3.5	-56.0 ± 1.9	-50.3 ± 1.8	148.0± 37.2

Table S4. Chronic effects of AZM treatment on Ca^{2+} and Na^{+} currents in iPSC-CMs

Ionic currents	Concentrations (µM)	Peak current density (pA/pF)	Cell capacitance (pF)	Steady-state activation		Steady-state inactivation	
				$V_{1/2}$ (mV)	k	$V_{1/2}$ (mV)	k
**Ca^{2+} currents**	0	-29.5 ± 2.1	57.1 ± 4.4	-15.2 ± 0.5	3.8 ± 0.3	-27.8 ± 0.6	5.2 ± 0.1
**At 0 mV, n= 9-16**	30	-14.9 ± 1.0	55.4 ± 2.9	-13.1 ± 0.9	5.1 ± 0.4	-26.5 ± 1.0	5.5 ± 0.2
**Na^{+} currents**	0	-100.8 ± 8.3	57.1 ± 4.4	-37.5 ± 1.1	3.3 ± 0.3	-71.6 ± 1.2	6.2 ± 0.2
**At -30 mV, n= 11-22**	30	-126.5 ± 8.1	55.4 ± 2.9	-35.5 ± 0.8	3.1 ± 0.2	-68.1 ± 1.2	5.2 ± 0.2
